# Calcium controls type III secretion switch through an SctV-SctW interplay

**DOI:** 10.3389/fmicb.2026.1800077

**Published:** 2026-05-19

**Authors:** Athina G. Portaliou, Pritam Roy, Rinky Parakra, Ana-Nicoleta Bondar, Spyridoula Karamanou, Anastassios Economou

**Affiliations:** 1 Laboratory of Molecular Bacteriology, Department of Microbiology, Immunology and Transplantation, Rega Institute for Medical Research, KU Leuven, Leuven, Belgium; 2 Faculty for Interdisciplinary Sciences, University of Bucharest, Bucharest, Romania; 3 Institute for Computational Biomedicine (INM-9), Forschungszentrum Jülich, Jülich, Germany

**Keywords:** Ca^2+^ regulation, SctV, SctW, secretion hierarchy, secretion switch, T3SS

## Abstract

Type III secretion (T3S) is employed by many Gram-negative pathogens to inject toxins into eukaryotic cells via a syringe-like nanomachine, the injectisome. Secretion of T3 substrates follows a hierarchical order: early substrates and translocators are secreted first to assemble the injectisome, followed by effectors that initiate infection. Gatekeeper proteins and environmental signals, such as calcium, are known to regulate the secretion switch from translocators to effectors; however, the underlying molecular mechanism remains elusive. In this study, we demonstrate that the gatekeeper SctW and the export apparatus component SctV bind Ca^2+^ in solution with high affinity. Ca-binding is not required for anchoring SctW to the export apparatus but modulates the affinity of this interaction. High-affinity, Ca-dependent association engages the SctV C-domain, promoting high-affinity targeting and secretion of translocators. Low Ca or loss of Ca-binding weakens the SctW–SctV association, leaving the SctV C-domain available for high-affinity effector targeting and secretion. Mutations in either component freeze the bipartite receptor in high or low-affinity states, thereby disrupting Ca-dependent crosstalk and blocking secretion. These findings indicate that calcium acts as a ruling factor of T3 secretion.

## Introduction

**Figure 1:**
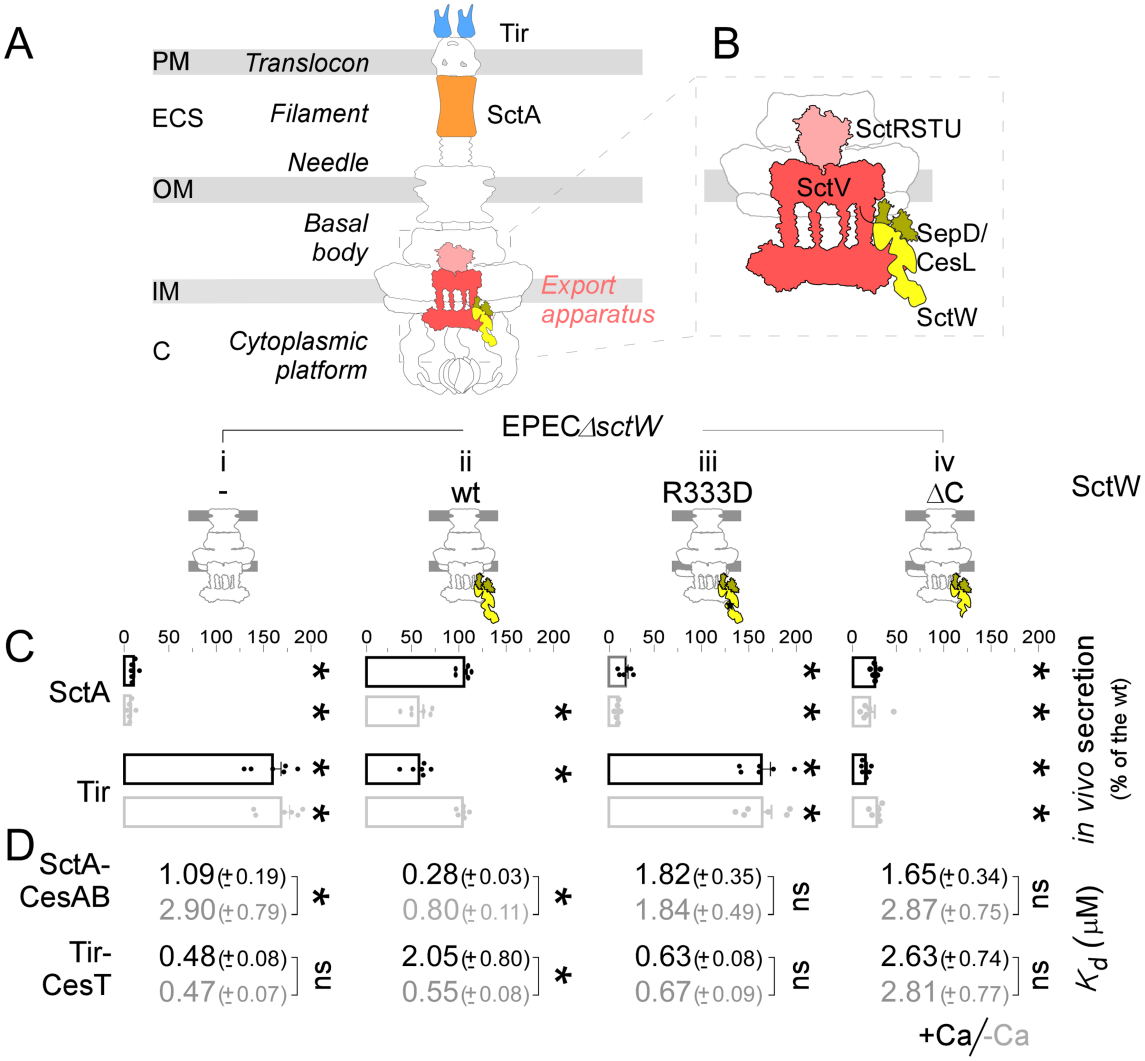
A functional SctW is required for the Ca regulation of T3 secretion. **(A,B)** Cartoon representation of the T3SS_EPEC_ injectisome **(A)** (Portaliou et al., 2016), with the export apparatus and associated gatekeeper complex magnified **(B)**. Protein names are based on the unified nomenclature (Schubot et al., 2005) whenever applicable. Red/pink: export apparatus; yellow: gatekeeper complex; blue: effector (Tir); orange: translocator (SctA). IM: Inner membrane; OM: Outer membrane; ECS: extracellular space; PM: Host plasma membrane. **(C)** Quantification of the *in vivo* SctA and Tir secretion (as indicated) by EPECΔ*sctW* complemented *in trans* with *sctW*, or the indicated derivatives, under − (gray)/+ (black) calcium (Ca) conditions. A representative experiment is shown in Supplementary Figure S1A. The SctA amount secreted from cells complemented with wild-type *sctW* in the + Ca condition/Tir amount secreted from cells complemented with wild-type *sctW* in the -Ca condition were set as 100%; all other SctA/Tir values were expressed relative to these values. *n* ≥ 3 independent repeats and mean values with SD are shown. Statistical significance, relative to 100% controls, is indicated with an asterisk (*p*-values < 0.01); non-significant (ns). **(D)** Apparent dissociation constants (*K*_d_) of translocator-chaperone (SctA-CesAB) and effector-chaperone (Tir-CesT) complexes for IMVs generated from EPEC*sctW* cells complemented *in trans* with *sctW* or the indicated derivatives, under − (gray)/+ (black) Ca conditions. Data were analyzed by non-linear regression (GraphPad; Prism); average values with SEM are shown. *n* = 6 independent repeats. Statistical significance between −/+ Ca conditions is indicated with an asterisk (*p*-values < 0.001); non-significant (ns).

Type III secretion systems (T3SSs) are major virulence determinants in many Gram-negative bacterial pathogens that are used to inject toxins into the eukaryotic cytoplasm (Sanchez-Garrido et al., 2022; Portaliou et al., 2016). More than 20 proteins coordinately assemble into a complex, trans-envelope, syringe-like structure, the injectisome (Figure 1A; Portaliou et al., 2016; Manisha et al., 2024). The SctR-S-T-U-V export apparatus pre-assembles at the base of the basal body (Figures 1A,B) and is responsible for substrate sorting, targeting, and translocation across the inner membrane (Lara-Tejero et al., 2011; Wagner et al., 2018; Portaliou et al., 2017). Its major component, SctV, self-assembles into an inner membrane nonameric ring structure (Yuan et al., 2021; Matthews-Palmer et al., 2021; Kuhlen et al., 2021; Majewski et al., 2020), with a cytoplasmic domain that serves as a docking site for chaperones, T3S substrates, and other cytoplasmic components (Figure 1B, red; Yuan et al., 2021; Matthews-Palmer et al., 2021; Rossi et al., 2023; Yu et al., 2018). SctR-S-T-U are arranged in a conical structure (Johnson et al., 2019; Kuhlen et al., 2018) (Figure 1B, pink) on top of the SctV ring, providing the structural template for the assembly of the rod–needle complex (see below) (Hu et al., 2018). At the base of the injectisome, the highly dynamic cytoplasmic ring (SctQ- K) and ATPase complex (SctL-N-O) interact with the inner ring (SctD) and SctV to form a cytoplasmic platform (Wimmi et al., 2024; Jensen et al., 2020; Shen and Blocker, 2016; Romo-Castillo et al., 2014; Brianceau et al., 2025; Figure 1A).

The remaining injectisome is built by T3S substrates (Figure 1A). Their secretion through the export apparatus follows a strict hierarchy (Portaliou et al., 2017; Kuhlen et al., 2018), and they are classified as follows: (i) “Early substrates”; SctI and SctF form the inner rod and needle. Formation of the needle triggers conformational changes at the export apparatus (Yu et al., 2018; Kuhlen et al., 2020; Monjaras Feria et al., 2015), which may involve SctU auto-cleavage and lead to secretion switches to that of translocators (Kuhlen et al., 2020; Monjaras Feria et al., 2015). (ii) “Middle substrates” (hereafter “translocators”); SctA forms the filament at the tip of the needle, and SctB and SctE form the translocation pore. Embedment of the translocator pore into the host plasma membrane marks the completion of the injectisome and signals the switch to effectors/toxins secretion (Sanchez-Garrido et al., 2022; Portaliou et al., 2016). (iii) “Late substrates” (hereafter “effectors”) are bacterium-specific (Manisha et al., 2024), and once injected into the host cytoplasm, they mediate cellular processes that lead to host cell death (Portaliou et al., 2016; Deng et al., 2017).

The cytoplasmic SctW protein, known as the gatekeeper, prevents premature effector secretion (Gaytan et al., 2018; Burkinshaw et al., 2015; Younis et al., 2010) by using diverse mechanisms across different bacteria (Yu et al., 2018; Botteaux et al., 2009; Pienkoss et al., 2021). SctW comprises a highly disordered N-terminal region, which binds the heterodimer chaperone complex SepD-CesL in enteropathogenic *Escherichia coli* (EPEC) (Gaytan et al., 2018; Burkinshaw et al., 2015), and three helical C-terminal domains connected with hinges (Burkinshaw et al., 2015). Gatekeepers are highly conserved among T3SS pathogens, and the crystal structures from *Chlamydia*, *Shigella*, *Yersinia,* and *EPEC* are almost identical (Burkinshaw et al., 2015; Nawrotek et al., 2014; Deane et al., 2008; Schubot et al., 2005). SctW may associate with the injectisome by binding to the SctV ring (Figure 1B; Portaliou et al., 2017; Yu et al., 2018), but it has never been resolved as an injectisome component, likely suggesting low abundance or/and an easily detachable complex (Berger et al., 2021; Miletic et al., 2020; Hu et al., 2019; Hu et al., 2017).

Environmental factors, such as pH and calcium (Ca), can trigger secretion switching from translocators to effectors (Manisha et al., 2024; Yu et al., 2010; Dickenson et al., 2010). Although not abundant in human cells (~120 nM intracellular Ca^2+^), Ca is abundant in the extracellular fluid (~2.5 mM Ca^2+^) (Jairaman and Prakriya, 2024; Lee and Auersperg, 1980) and has been implicated in virulence (Dominguez et al., 2015); an ion influx into the bacterial cytoplasm is believed to occur upon completion of the needle. While in some bacteria, Ca depletion enhances injectisome production [*Yersinia* (Straley et al., 1993); *Pseudomonas aeruginosa* (Dasgupta et al., 2004)], in EPEC Ca promotes translocator secretion (SctA/B/E) with no effect on injectisome assembly (Gaytan et al., 2018; Deng et al., 2005).

The molecular mechanism by which Ca switches secretion is unknown. Various sources of evidence suggest that the gatekeeper is also implicated in this mechanism (Younis et al., 2010; Deng et al., 2005; Shaulov et al., 2017). The gatekeeper itself acts as a secretion switch (Portaliou et al., 2016; Portaliou et al., 2017; Gaytan et al., 2018; O'Connell et al., 2004); deletion of *sctW*, or its chaperones, leads to cells that can no longer switch secretion from translocators to effectors (Gaytan et al., 2018; Deng et al., 2005; O'Connell et al., 2004). The possibility that both the gatekeeper SctW (the primary receptor for translocators) (Portaliou et al., 2017) and the major component of the export apparatus SctV (the main receptor for effectors) (Yuan et al., 2021) are implicated in the Ca-dependent secretion switch process has not been excluded. We tested this hypothesis by probing the Ca interaction with SctW or SctV, *in vivo* and *in vitro*, using mutagenesis, secretion assays, membrane affinity measurements, isothermal titration calorimetry (ITC), and hydrogen deuterium exchange-mass spectrometry (HDX-MS) experiments. In this study, we demonstrate that both SctW and SctV bind calcium in solution and demonstrate that a crosstalk between Ca, SctW, and SctV dictates the affinity of the gatekeeper and secretory proteins for their receptor(s), thereby determining secretion hierarchy.

## Materials and methods

### *In vivo* secretion assays

EPEC wild-type (wt) or deletion strains carrying, *in trans*, the indicated plasmids were grown overnight at 37 °C in Luria broth (LB) supplemented with 100 μgr/mL ampicillin. Cells were diluted 1:100 in 50 mL optimized M9 medium (Yuan et al., 2018), supplemented with 100 μgr/mL ampicillin, 2.5 mM CaCl_2_ (+Ca) or 0.1 mM EGTA (−Ca), and grown at 37 °C under shaking conditions. At OD_600_ = 0.3, AHT was added to induce the expression of *sctV* or *sctW* (2.5 ng/mL or 5 ng/mL, respectively) and their derivatives. Cells were then grown for an additional 3 h. Once the OD_600_ values of the cultures were recorded, cells were separated from the spent growth medium by centrifugation (4,000×*g*; 20 min; 4 °C). Using the OD_600_ values, the spent medium from different cultures/strains was normalized to 0.5 mL wt EPEC culture at OD_600_ = 0.3. The corresponding samples were filtered using a vacuum manifold (Bio-Dot apparatus; Bio-Rad) through nitrocellulose in order to immobilize secreted proteins from the spent growth medium onto the membrane, and then, a western blot analysis was performed. Secretion of SctA and Tir was monitored using protein-specific rabbit polyclonal antibodies. Signals were visualized and quantified using a Las4000 ImageQuant (GE Healthcare) and ImageQuant TL software (GE Healthcare). SctA signals (−/+Ca) were normalized and plotted as a percentage of the +Ca-wild-type condition; Tir signals (−/+Ca) were normalized and plotted as a percentage of the −Ca-wild-type condition.

### Protein purification

As previously described (Portaliou et al., 2017; Yuan et al., 2021), all proteins or protein complexes were overproduced in *E. coli* BL21 (DE3) or C-41 (DE3) cells under the control of the T7 promoter (LB medium; 37 °C; 0.01 mM IPTG; 3 h induction). Bacterial cells from 15-L cultures were harvested by centrifugation (4,500×*g*; 4 °C; 15 min; Avanti J-26S XPI, JLA 8.1000 rotor; Beckman) and stored at −20 °C until purification. Cells were solubilized in buffer E, supplemented with 50 μg/mL DNase I and 2.5 mM PMSF, and lysed using a French press (1,000 psi; 5 rounds; Thermo Scientific). Unbroken cells and the insoluble fraction were removed by centrifugation (26,600×*g*; 30 min; 4 °C, Sorvall RC 6 plus, Fisher Scientific). The soluble fraction containing the protein of interest was loaded onto a home-made, Ni^2^-NTA Agarose (Qiagen) column that was pre-equilibrated with buffer E using gravity flow (1 mL/min). The column was washed sequentially with buffers E/F (10 column volumes each), and proteins were eluted with buffer F supplemented with 300 mM imidazole. Eluted proteins were subjected to size exclusion chromatography using a Superose™6 10/300 GL column (GE Healthcare), pre-equilibrated in Buffer A, on an ÄKTA Pure system (GE Healthcare). Peak fractions were collected and concentrated using spin columns (Amicon® Ultra Centrifugal Filter, 30 kDa MWCO; Merck). Purified proteins were dialyzed in Buffer C (Medicell Membranes Ltd.; overnight, 4 °C), aliquoted, and stored at −20 °C until further usage. Protein purity was determined on SDS–PAGE coupled with Coomassie staining.

### Protein concentration

Protein concentration was determined spectroscopically (280 nm; Nanodrop 2000; Thermo) in the 0.3–3 mg/mL range, using individual molecular weights and extinction coefficients calculated with the ExPASy server.^1^ To concentrate protein samples, centrifugal ultrafiltration concentrators were used [10–30 kDa CO; Vivaspin 500 (12,000×*g*; 4 °C) or Vivaspin 4 (4,500×*g*; 4 °C); Sartorius].

### Preparation of inverted inner membrane vesicles (IMVs)

IMVs were prepared from *E. coli* C41, EPEC, and derivative strains (as indicated) as previously described (Portaliou et al., 2017; Cunningham et al., 1989; Lill et al., 1989). Briefly, cells grown overnight (300 mL LB; 37 °C) were used to inoculate 15 L of LB (37 °C; under shaking conditions). Following 3 h growth, the expression of *sctV* or *sctW*, or their derivatives, was induced (2.5 ng/mL or 5 ng/mL AHT, respectively), and cells continued growing for an additional 3 h before harvesting by centrifugation (5,000×*g*; 20 min; 4 °C; Avanti J-26S XPI, Beckman; JLA 8.1000 rotor). Cell pellet was resuspended in Buffer A supplemented with 20% glycerol and lysed using a French press (8,000 psi; 5–6 passages; Thermo Scientific). Unbroken cells were removed by centrifugation (3,000×*g*; 10 min; 4 °C; Sigma 3-16KL; rotor 11180), and the supernatant was further ultracentrifuged (100,000×*g*; 90 min; 4 °C; fixed angle 45Ti rotor; Optima XPN-80, Beckman). The membrane pellet was resuspended in Buffer A containing 20% glycerol using a Dounce homogenizer. Then, 2.5 mL membrane resuspension was loaded onto a five-step sucrose gradient (from bottom to top: 1.9; 1.7; 1.5; 1.3; 1.1 M sucrose in 50 mM Tris pH: 8.0; 6 mL/layer) and centrifuged (100,000×*g*; 16 h; 4 °C; swinging bucket SW32 Ti rotor, OptimaXPN-80, Beckman; equilibrium centrifugation). Inverted Inner Membrane Vesicles (IMVs) were collected from gradient fractions 2–3 and re-centrifuged to pellet them down (100,000×*g*; 90 min; 4 °C; fixed angle 45Ti rotor; Optima XPN-80, Beckman). The membrane pellet was resuspended in 6 M urea alongside Buffer B using a Dounce-homogenizer and incubated for 35 min on ice. This material was loaded on top of an equal volume of 0.2 M Sucrose-Buffer B and centrifuged (sucrose cushion; 100,000×*g*; 90 min; 4 °C; swinging bucket SW32 Ti rotor, Optima XPN-80, Beckman). Finally, IMVs were collected, resuspended in Buffer B, homogenized by passing through an Avestin LiposoFast-Basic system (100 nm pore size filter; 15 times), and stored in aliquots at −80 °C.

### Determination of equilibrium dissociation constants (apparent *K*_d_)

Equilibrium dissociation constants were determined as previously described (Portaliou et al., 2017; Gouridis et al., 2009).

*Protein labeling:* For ^35^S-labeling of proteins, Easy TagTM L-[^35^S]-methionine (1 mC; Perkin Elmer) and the TNT® Quick coupled Transcription/Translation systems (Promega) were used according to the manufacturer’s instructions. Non-incorporated [^35^S]-methionine was removed during a buffer exchange step using home-made spin columns of 1 mL G-50 resin pre-equilibrated with Buffer A. Labeled proteins were aliquoted and stored in Buffer A containing 10% glycerol (maximum 3–5 days;- 20 °C). *Membrane binding assay:* 4 μM of unlabeled purified proteins/complexes were treated with 0.1 mM EGTA (30 min; 4 °C) and serially diluted in Buffer B supplemented with 1 mg/mL BSA and either 2.5 mM CaCl_2_ or 0.1 mM EGTA (+/−Ca condition, respectively). Twenty different protein concentrations ranging from 0.01 to 1 μM were mixed with a specific amount of IMVs (20 μg total membrane protein/reaction; 20 μL in Buffer B) and 1 μL of ^35^S-labeled protein (same as the unlabeled; used as tracer). Samples were incubated (20 min; ice), overlaid into an equal volume of sucrose cushion buffer (0.2 M Sucrose; 1 mg/mL BSA in Buffer B), and centrifuged (300,000×*g*; 20 min; 4 °C; rotor TLA-100; Optima Max-XP, Beckman-Coulter) to separate unbound proteins from IMVs. The pellet (containing IMVs and IMV-bound proteins) was resuspended in 300 μL Buffer B using a water-bath sonicator (Branson) and filtered through a nitrocellulose membrane using a vacuum manifold (Bio-Dot apparatus; Bio-Rad) to immobilize proteins. IMV-bound protein ^35^S-signals were visualized by autoradiography using a high-resolution phosphor storage screen (GE Healthcare) and scanned on a Typhoon FLA 9500 system (GE Healthcare; default settings). Protein signals were quantified using Image Quant software (GE Healthcare) and normalized against the total amount of ^35^S-labeled protein used per reaction. Normalized IMV-bound protein values were plotted against the total protein amount used per reaction. Data were analyzed by non-linear regression fit for one binding site, using Prism 5.0 (GraphPad). For the determination of an apparent *K*_d_, a set of 20 concentration points was used per dataset, with each set repeated at least six times independently.

### Isothermal titration calorimetry (ITC)

Protein samples were extensively dialyzed (100× sample volume) in Buffer A supplemented with 0.1 mM EGTA (overnight; 4 °C; constant stirring). To remove excess EGTA, we additionally performed two sequential dialysis steps in Buffer A (1 h; 4 °C; constant stirring). The experiments were performed using a MicroCal iTC_200_ System (GE Healthcare). The cell was filled with the indicated protein or complex (10 μM; 300 μL buffer A) and the syringe with CaCl_2_ (150 μM; 100 μL buffer A). For the CaCl_2_ titration, the experimental setup consisted of 2 μL injections at 4-min intervals, with a cell temperature of 25 °C and constant stirring at 300 rpm. Data were analyzed using MicroCal Origin software version 7.0 (GE Healthcare).

### Local hydrogen-deuterium exchange (HDX) mass spectrometry (MS)

*Sample preparation*: Prior to HDX-MS experiments, proteins were further purified using size-exclusion chromatography coupled online to multi-angle light scattering (SEC-MALS; Superdex200 10/300; GE HealthCare). Approximately 150 μM protein (500 μL; Buffer D) was incubated with 5 mM DTT (30 min; ice), centrifuged to remove protein aggregates (20,000×*g*; 10 min, 4 °C), and injected into SEC-MALS. Protein peak fractions were collected, concentrated (up to ~200 μM), treated with 0.1 mM EGTA (30 min; ice), and divided into two parts. In one part, 5 mM CaCl_2_ (+Ca condition) was added, and to the other, equal volume of buffer D (−Ca condition). *D_2_O-labeling:* Lyophilized aliquots of Buffer D were solubilized in D_2_O (99.9% atom D, Sigma Aldrich P/N 151882). For isotope labeling, 10–20 μM protein was incubated in D_2_O buffer for the indicated amount of time (90%; 25 °C; 10 s, 1 min, 10 min, 100 min) at 30 °C. The exchange reaction was quenched by the addition of pre-chilled Quench buffer at a 1:1 ratio (final pH: 2.3), incubated during a centrifugation step (20,000×*g*; 2 min; 4 °C), and injected into the instrument. *Controls:* Non-deuterated samples for peptide mass identification and fully deuterated (FD; 100% D-uptake) samples were used as controls. For FD samples, the purified protein was incubated in D_2_O buffer for 14 h (gatekeeper complex) or 72 h (SctV-CD) at 30 °C, quenched, and treated as all other samples. *MS analysis:* Samples were injected manually. Peptides were generated using an immobilized Nepenthesin-2 cartridge (2.1 × 20 mm; Affipro) at 16 °C. The UPLC chamber was set at 2 °C to avoid back-exchange. Peptides were trapped onto a VanGuard C_18_ pre-column (130 A, 1.7 mm, 2.1 × 5 mm, Waters) at 100 μL/min for 3 min, using ddH_2_O–0.23% (v/v) formic acid, and separated on a C_18_ analytical column (130 A, 1.7 mm, 1 × 100 mm, Waters) at 40 μL/min. UPLC separation (solvent A: 0.23% v/v formic acid, solvent B: 0.23% v/v formic acid in acetonitrile) was performed using a 12-min linear gradient (5–50% solvent B). Solvent B was then raised to 90% for 1 min to wash out remaining proteins. The peptide spectrum of the unlabeled protein had been first determined using the same conditions. *Peptide identification and data analysis*: ProteinLynx Global Server (PLGS v3.0.1, Waters, UK) and the primary sequences of SctV, SctW, SepD, and CesL were used. Peptides were individually inspected; only those with a signal-to-noise ratio of >10, PLGS score of >7, and detected in >3 repeats were included in the analysis. For raw mass spectral data compilation and calculation of relative deuteration from centroid values, DynamX 3.0 (Waters, Milford, MA, USA) software was used. *Quantification and analysis of HDX-MS data*: D-exchange experiments were carried out in at least three replicates across multiple days. All spectra were manually curated. Deuterium uptake values were exported from DynamX as ‘state files’ (.csv) and are shown in Supplementary Table 1.

### Statistics/reproducibility

Data points in secretion plots represent measurements from independent biological repeats over multiple days, and are shown with ± SD. *K*_d_ values are reported as best-fit values ± SEM, with 95% CI (Prism 5.0; GraphPad). Statistical analysis was performed to determine significant differences within our data using the paired T-test analysis (Prism 5.0; GraphPad). *Secretion experiments*: for *p* values < 0.01, one asterisk is used. *Affinity measurements:* for *p* values <0.001, one asterisk is used. Values larger than the indicated *p* values were considered non-significant (ns).

### Miscellaneous

Buffers (Supplementary Table S2), antisera (Supplementary Table S3), bacterial strains (Supplementary Table S4), vectors and constructs (Supplementary Table S5), and primers (Supplementary Table S6) used in the study are listed in Supplementary material.

## Results

### A functional SctW is required for the Ca-dependent regulation of T3 secretion

To dissect the molecular determinants of the SctW–Ca crosstalk, we used two known, non-functional SctW derivatives: (i) SctW(R333D): the conserved R333 is essential for translocator secretion (Gaytan et al., 2018; Burkinshaw et al., 2015; Barkalita et al., 2020) and (ii) SctW(ΔC): deletion of the last ten C-terminal residues causes defective translocator secretion (Wang et al., 2008).

EPECΔ*sctW* cells were transformed with plasmids carrying either *sctW*, *sctW*(R333D), or *sctW*(ΔC). Cells were grown in M9 medium (Yuan et al., 2021; Yuan et al., 2018) in the presence of 2.5 mM CaCl_2_ or 0.1 mM EGTA (hereafter +/− Ca conditions, respectively). Secreted proteins were immobilized on nitrocellulose using a vacuum manifold to filter spent medium (0.5 mL OD_600_ = 0.3 culture). SctA or Tir (translocator/effector, respectively) were immunodetected using protein-specific antibodies (a representative experiment is shown in Supplementary Figure S1A), and signals were quantified. EPECΔ*sctW* cannot secrete translocators; instead, it secretes effectors in any Ca condition (Figure 1C, i). Plasmid-born *sctW* is sufficient to restore Ca-regulated secretion for both effectors and translocators (ii), that is, Tir is upregulated/downregulated in −/+ Ca conditions respectively, whereas SctA exhibits the opposite regulation, being upregulated/downregulated in +/− Ca conditions respectively. *sctW*(R333D) cannot restore translocators secretion (iii; compare to i), in agreement with previous studies (Barkalita et al., 2020; Wang et al., 2008). *sctW*(ΔC) is unable to restore the secretion of translocators and additionally inhibits effector secretion (iv).

Summarizing, T3SS exhibits differential secretion of translocators/effectors in a Ca-dependent manner. To retain this regulation, a functional SctW is needed.

### SctW–Ca crosstalk dictates the affinity of T3S substrates for their receptors

Using an *in vitro* membrane-binding assay, we had previously established that SctW binding to SctV regulates the relative affinities of T3S substrates for the export apparatus (Portaliou et al., 2017). We used the same assay to examine whether calcium affects this affinity. To this end, inverted membrane vesicles (IMVs) were prepared from EPECΔ*sctW* strains complemented with either an empty plasmid or plasmids carrying either *sctW*, *sctW*(R333D), or *sctW*(ΔC). A fixed amount of IMVs was incubated with various concentrations of purified secretory protein–chaperone complexes, together with a fixed amount of ^35^S-labeled ones used as tracer (SctA-CesAB or Tir-CesT, 20 min, ice). The amount of protein complexes that remained bound to IMVs following ultracentrifugation through a sucrose cushion was visualized by autoradiography, quantified, plotted against the total amount added, and helped determine the affinity of the complex for IMVs.

In the absence of SctW, regardless of Ca condition, translocators are bound to IMVs with a *K*_d_ ~ 1–3 μM (Figures 1D, i) and effectors to IMVs with a *K*_d_ ~ 0.4 μM. In the presence of SctW, in the +Ca condition, the affinity for translocators increases to ~0.3 μM (ii, black), whereas the affinity for effectors decreases to ~2 μM. In the −Ca condition (gray), the opposite effect is observed; the affinity for translocators decreases to 0.8 μM and that of effectors increases to 0.55 μM. SctW(R333D)-IMVs demonstrate translocator and effector binding that is similar to the one seen for Δ*sctW*-IMVs, with no distinction between −/+ Ca conditions (compare iii to i). SctW(ΔC)-IMVs exhibit equally low affinity binding for either translocators or effectors under any Ca condition (iv). Thus, mutations in SctW affect the affinity of clients for their receptors as well as the secretion switch.

Our results demonstrate that SctW differentially regulates the relative affinities of T3S substrates in a Ca-dependent manner, suggesting a direct SctW–Ca crosstalk.

### The SctW-SepD-CesL complex binds Ca in solution

Next, we investigated whether SctW can bind Ca. *In silico* analysis using CalPred^2^ indicated that SctW, SepD, and CesL do not contain canonical Ca-binding motifs. On the other hand, molecular dynamics (MD) simulations showed that SctW can bind Ca in solution at multiple, non-canonical binding sites (Supplementary Figure S1B).

**Figure 2:**
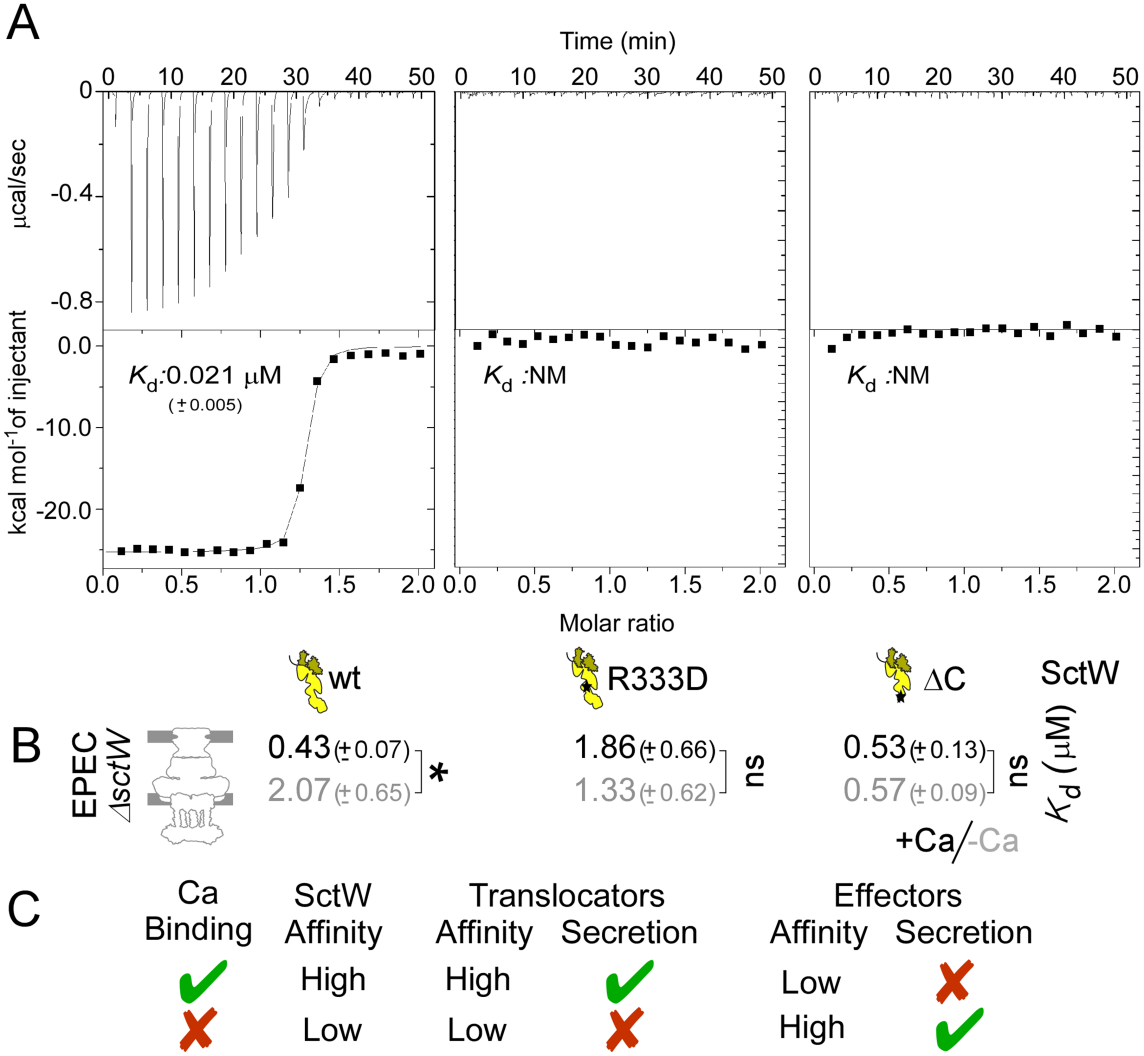
Calcium binding by SctW determines the affinity of its membrane association. **(A)** Apparent dissociation constants (*K*_d_) of the SctW-SepD-CesL complex, or the indicated derivatives, for calcium in solution, by ITC experiments. NM: non-measurable. *n* = 3 independent repeats. **(B)** Apparent dissociation constants (*K*_d_) of the SctW-SepD-CesL complex, or the indicated derivatives, for IMVs generated from *EPEC*ΔsctW cells, under − (gray)/+ (black) Ca conditions. Data were analyzed by non-linear regression (GraphPad; Prism); average values with SEM are shown. *n* = 6 independent repeats. Statistical significance between −/+ Ca conditions is indicated with an asterisk (*p*-values < 0.001); non-significant (ns). **(C)** Summary of the secretion switch mechanism in T3SS of EPEC.

To directly probe into this, we employed isothermal titration calorimetry (hereafter ITC). SctW, which cannot remain soluble in the absence of its cognate chaperones (Gaytan et al., 2018; Burkinshaw et al., 2015), was purified in complex with the CesL and SepD chaperones. Mutations in SctW did not affect the gatekeeper complex formation (Supplementary Figure S1C). A total of 150 μM of CaCl_2_ in buffer A was titrated into a cell containing 10 μM SctW-SepD-CesL complex in the same buffer at 25 °C. A clear binding isotherm with a dissociation constant *K*_d_ ~ 0.02 μM was observed (Figure 2A). Under identical experimental conditions, neither SctW(R333D)-SepD-CesL nor SctW(ΔC)-SepD-CesL displayed measurable Ca binding.

Clearly, the gatekeeper complex can bind Ca in solution with high affinity. The fact that Ca binding is abolished by two different mutations in the C-terminal region of SctW suggests a likely binding site in the region. Assuming a Ca-binding site on SepD or CesL, which is destroyed through long-range allosteric effects by two different SctW mutations on the other end of the chaperone binding sites, cannot be excluded but seems unlikely.

### The gatekeeper affinity for its receptor is modulated by Ca

To examine whether the interaction with Ca affects the gatekeeper’s affinity for its receptor, we used an *in vitro* membrane-binding assay. IMVs were prepared from EPECΔ*sctW* under Ca-replete and Ca-depleted conditions. A fixed amount of IMVs was incubated with various concentrations of a purified SctW–SepD–CesL complex that was mixed with a fixed amount of S^35^-labeled complex (used as tracer) under −/+ Ca conditions. Similar experiments were repeated using the SctW(R333D)–SepD–CesL and SctW(ΔC)–SepD–CesL complexes. The affinity of gatekeeper complexes for EPECΔ*sctW* IMVs was determined (as in Figure 1D). The wild-type gatekeeper complex binds IMVs with a *K*_d_ ~ 0.4 μM in the +Ca condition (Figure 2B); this affinity decreases to ~2 μM in the −Ca condition. The SctW(R333D) complex demonstrated a *K*_d_ ~ 1.5 μM for the same IMVs, whereas the SctW(ΔC) complex demonstrated a *K*_d_ ~ 0.5 μM. Neither mutant is affected by Ca.

In summary (Figure 2C), Ca binding to the gatekeeper complex promotes higher-affinity binding at the membrane, which leads to higher-affinity binding/secretion of translocators and lower-affinity binding/no secretion of effectors. Lack of Ca binding on SctW dictates lower-affinity association with the membrane, which in turn results in lower-affinity binding/no-secretion of translocators and higher-affinity binding/secretion of effectors. The SctW(R333D) phenotype is fully consistent with this model (Figures 2B, 1C,D, iii). Deletion of the C-tail, SctW(ΔC), induces a gatekeeper conformation that is compatible with increased affinity binding, despite the lack of Ca-binding (Figures 2A,B). Nevertheless, as the C-domain of SctW is required for SctA-CesAB binding (Portaliou et al., 2017), SctW(ΔC) fails to support higher affinity binding/downstream secretion of translocators (Figures 1C,D, iv).

### SctV is necessary, but not sufficient, for higher-affinity-Ca-regulated SctW binding

SctW is thought to guide secretory substrates to the export apparatus through its association with SctV (Portaliou et al., 2017; Gaytan et al., 2018). Participation of additional injectisome components, such as the cytoplasmic domain of SctU or the molecular ruler SctP (Shaulov et al., 2017; Marcos-Vilchis et al., 2025), has not been excluded.

**Figure 3:**
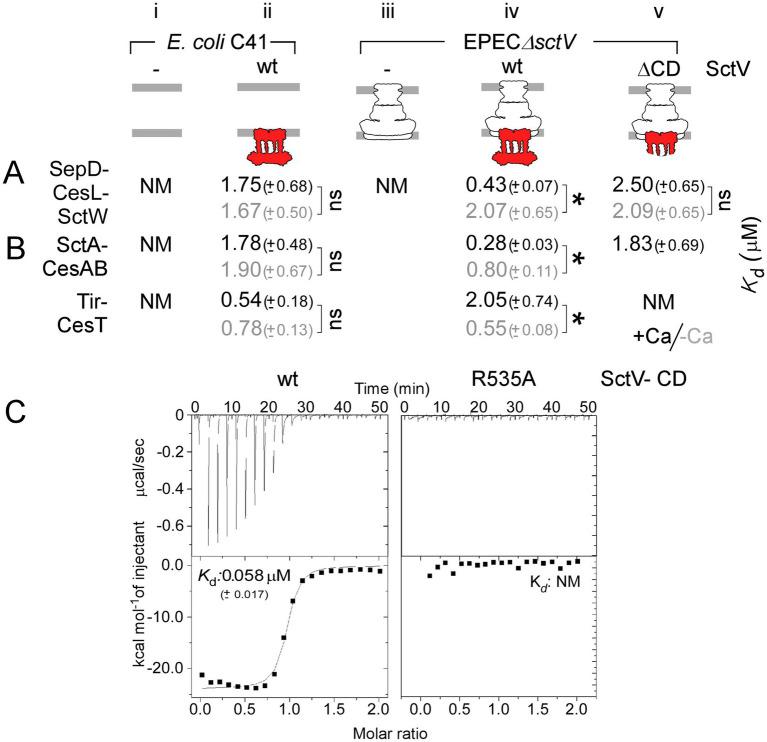
SctV is necessary but not sufficient for high-affinity-Ca-regulated SctW and translocator binding. **(A,B)** Apparent equilibrium dissociation constants (*K*_d_) of the gatekeeper (SctW-SepD-CesL; **A**), translocator-chaperone (SctA-CesAB; **B**), and effector-chaperone (Tir-CesT; **B**) complexes for IMVs generated from *E. coli* C41 or EPECΔ*sctV* cells carrying *in trans* sct*V*, or the indicated derivatives, under − (gray)/+ (black) Ca conditions. Data were analyzed by non-linear regression (GraphPad; Prism); average values with SEM are shown. NM: non-measurable. *n* = 6 independent repeats. Statistical significance between −/+ Ca conditions is indicated with an asterisk (*p*-values < 0.001); non-significant (ns). **(C)** Apparent dissociation constants (*K*_d_) of the SctV C-domain (SctV-C), or the indicated derivatives, for calcium in solution, by ITC experiments. NM: non-measurable. *n* = 3 independent repeats.

We probed into this using our *in vitro* membrane-binding assay. IMVs were prepared from *E. coli* C41 strain, which does not encode for a T3SS, and transformed with an empty plasmid or one carrying *sctV* from EPEC. A fixed amount of IMVs was incubated with various concentrations of SctW-SepD-CesL, and the affinity of the gatekeeper complex for those IMVs was determined under −/+ Ca conditions (as in Figure 1D). The gatekeeper has non-measurable affinity for C41-IMVs (NM; Figures 3A, i). In the presence of plasmid-born *sctV*, binding of SctW-SepD-CesL to IMVs occurs with a *K*_d_ ~ 1.7 μM, irrespective of the Ca− condition (ii). These results demonstrate that the gatekeeper complex does not bind non-specifically to lipids/membrane components of C41 and exhibits low-affinity binding, specifically for the SctV receptor, irrespective of the Ca condition.

The gatekeeper affinity was also measured using IMVs prepared from EPECΔ*sctV* cells that were transformed with an empty plasmid, or one carrying *sctV*, or *sctV*(ΔCD), where the cytoplasmic domain of SctV was deleted (Portaliou et al., 2017; Yuan et al., 2021). In the absence of SctV, there is no measurable affinity for the gatekeeper complex (NM; Figure 3A, iii), demonstrating that SctV is its sole receptor. Plasmid-born *sctV* restores the Ca-regulated gatekeeper binding (iv), in contrast to C41-S*ctV* IMVs (ii). Deletion of the SctV C-domain allows only for relatively low-affinity binding, in any Ca condition (v).

Our results provide evidence for a direct SctW-SctV interaction *in vitro*. SctV alone is necessary and sufficient for relatively low-affinity SctW binding (Figure 3A, i–ii) at the N-terminal part of the receptor (v); however, it is not sufficient for increased-affinity-Ca-regulated SctW binding. For the latter, SctV needs to be embedded in the injectisome (iv), and the SctV-C domain is required (v). Given that the gatekeeper alone can bind Ca with high affinity in solution (Figure 2A), the lack of Ca-regulated SctW binding in C41 or EPEC*sctV*(ΔCD)–IMVs argues for an additional level of regulation that is missing under these conditions.

### SctV is necessary but insufficient for Ca-dependent affinity translocator binding

To probe the requirements for increased-affinity substrate binding, we measured the affinity of translocator- or effector- chaperone complexes (SctA-CesAB and Tir-CesT, respectively) for C41-IMVs under −/+ Ca conditions (as in Figure 2B). Neither translocators nor effectors demonstrate measurable affinity for C41 IMVs (NM; Figures 3B, i). The mere presence of SctV, in the absence of other injectisome components, is sufficient to establish translocator binding of *K*_d_ ~ 1.8 μM and effector binding of *K*_d_ ~ 0.6 μM. Neither binding is affected by Ca.

Similar affinity measurements were performed using IMVs from EPECΔ*sctV* cells transformed with plasmids carrying either *sctV* or *sctV*(ΔCD). Wild-type SctV fully restored Ca-regulated-binding of both effectors and translocators (Figure 3B, iv). Deletion of only the C-domain of SctV abolished binding of effectors (NM; v) but could support binding of translocators with a *K*_d_ ~ 1.8 μM (v).

SctV alone is necessary and sufficient for Ca-independent, relatively high-effector/low-translocator affinity (Figure 3B, ii). For Ca-regulated translocator/effector binding, SctV needs to be embedded in the injectisome, and a wild-type gatekeeper complex has to be present (Figures 1D, 3B).

### SctV cytoplasmic domain binds calcium in solution

In the absence of the SctV cytoplasmic domain, the gatekeeper cannot acquire high-affinity binding to IMVs (Figure 3A, v). Since SctW-SepD-CesL can independently bind Ca (Figure 2C), the possibility of SctV also sensing Ca through its cytoplasmic domain was raised.

Although *in silico* analysis showed that SctV lacks canonical calcium-binding motifs (CalPred), MD simulations showed that the SctV-CD can bind Ca in solution at multiple non-canonical binding sites (Supplementary Figure S2A). We directly tested calcium binding on the purified SctV-CD by employing ITC. A total of 150 μM CaCl_2_ in buffer A was titrated into a cell that contained 10 μM SctV-CD in the same buffer at 25 °C. A clear binding isotherm with a dissociation constant *K*_d_ ~ 0.06 μM was observed for the wild-type SctV-CD (Figure 3C). Under identical experimental conditions, the C-domain of the oligomerization-defective mutant SctV(R535A) (Yuan et al., 2021) displayed no measurable calcium binding.

The C-domain of SctV binds calcium with high affinity. Ca-binding by both the gatekeeper and SctV may be required for high-affinity SctW-binding at the membrane.

### Mutations on SctV affect the Ca-dependent secretion switch

**Figure 4:**
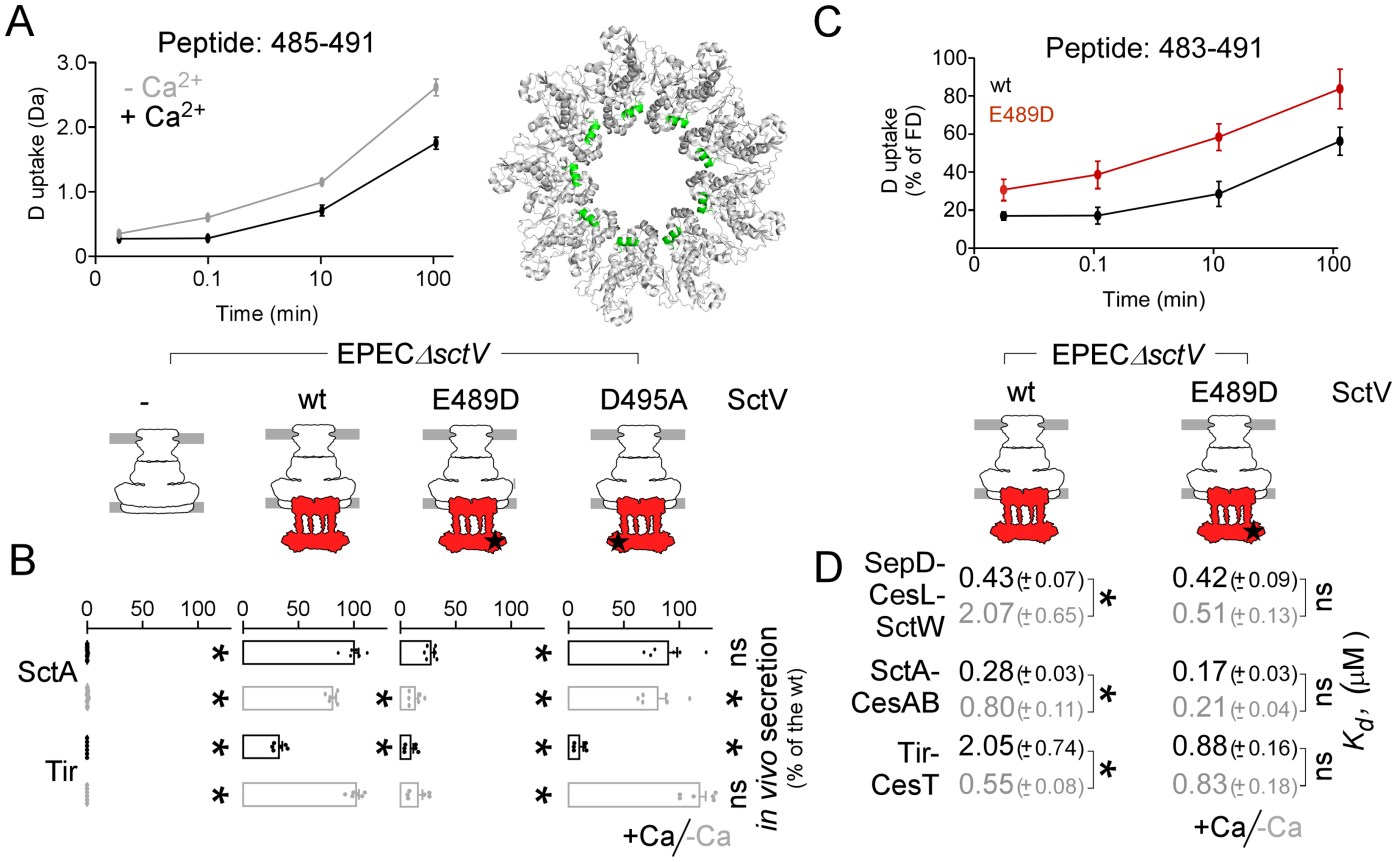
Mutations on SctV affect the Ca-dependent T3S switch. **(A)** Effect of calcium on the relative D-uptake of the indicated, representative of the region, SctV peptide, by HDX-MS experiments (left). *n* = 3 independent repeats; mean values with errors are shown. The 483–495 region is highlighted green on the SctV- CD_P7OSL_ structure (right). **(B)** Quantification of the *in vivo* SctA and Tir secretion (as indicated) by EPECΔ*sctV* cells complemented *in trans* with *sctV*, or the indicated derivatives, under − (gray)/+ (black) Ca conditions (as in Figure 1C). A representative experiment is shown in Supplementary Figure S2B. *n* ≥ 3 independent repeats and mean values with SD are shown. Statistical significance, relative to 100% controls, is indicated with an asterisk (*; *p*-values < 0.01); non-significant (ns). **(C)** Effect of the E489D mutation on the D-uptake of the indicated, region-representative SctV peptide, in the +Ca condition, by HDX-MS experiments. Values are shown as % D-uptake relative to the FD control (100%) of the same peptide. *n* = 3 independent repeats; mean values with errors are shown. **(D)** Apparent equilibrium dissociation constants (*K*_d_) of the gatekeeper (SctW-SepD-CesL), translocator-chaperone (SctA-CesAB) and effector-chaperone (Tir-CesT) complexes for IMVs prepared from EPECΔ*sctV* cells complemented *in trans* by *sctV*, or *sctV*(E489D), under − (gray) /+ (black) Ca conditions. Data were analyzed by non-linear regression (GraphPad; Prism); average values with SEM are shown. *n* = 6 independent repeats. Statistical significance between −/+ Ca conditions is indicated with an asterisk (*p*-values < 0.001); non-significant (ns).

Ca binding to either SctV or the gatekeeper may induce conformational changes that are sensed by the other partner. To test this hypothesis, the effect of Ca on the intrinsic dynamics of SctV–CD and SctW–SepD–CesL was monitored by local Hydrogen–Deuterium exchange Mass spectrometry (HDX-MS) (Wales and Engen, 2006). Briefly, 10–20 μM gatekeeper complex or SctV-CD in Buffer D supplemented with either 0.1 mM EGTA or 2.5 mM CaCl_2_ (−/+ Ca conditions respectively) was labeled in D_2_O (90%; 10 s, 1 min, 10 min, 100 min; 30 °C), quenched, protease-digested, and analyzed by MS. The D-uptake of peptides that covered the sequence length (>97% coverage) was determined (Supplementary Table S1). No significant difference was observed in the intrinsic dynamics of the gatekeeper complex between −/+ Ca conditions (Supplementary Table S1). With regard to the SctV-CD, we observed limited reduced dynamics in the +Ca-condition for the E483–D495 region that connects the D1–D3 domains (Figure 4A).

To test the role of this region, two *sctV* mutant derivatives were generated on conserved residues (E489D and D495A) (Majewski et al., 2020; Yu et al., 2018). EPECΔ*sctV* cells were transformed with an empty plasmid or plasmids carrying *sctV* or its mutant derivatives, and their ability to complement secretion *in vivo* under −/+ Ca was monitored by immunodetection of secreted proteins (as in Figure 1C). A representative experiment is shown in Supplementary Figure S2B. Mutant *sctV*(D495A) complemented secretion like wild type and was not followed further (Figure 4B). Mutant *sctV*(E489D) exhibited a major secretion defect, eliminating the secretion of both translocators and effectors. The C-domain of SctV(E489D) retained the wild-type oligomerization properties (Supplementary Figure S2C) and affinity for Ca binding in solution (Supplementary Figure S2D). HDX-MS experiments recorded significantly higher % D-uptake in the E483-D495 region for the SctV(E489)-CD, relative to that of the corresponding wild-type region, in the +Ca condition (Figure 4C). Destabilization of this region, despite Ca binding, suggested that the bound Ca might not be sensed correctly by SctV(E489D), leading to wrong signals concerning the secretion switch mechanism.

To test this, we determined the effect of this mutation on the affinity of the gatekeeper, translocators, and effectors for the export apparatus (as in Figures 3A,B). IMVs were generated from EPECΔ*sctV* cells that were complemented with plasmid-born *sctV* or *sctV*(E489D). We observed that the SctV(E489D) mutant appeared frozen in one conformation that supports relatively high-affinity binding for the gatekeeper, translocators, and effectors, with no distinction between −/+ Ca conditions (Figure 4D, compared to wild type under the same conditions).

Mutations in SctV disrupted the conformational cues needed to complete the secretion switch circuit, suggesting an active Ca-gatekeeper-SctV crosstalk.

## Discussion

Secretion of T3S substrates must comply with a strict hierarchical order to secure the sequential assembly of needles (by early substrates), filaments, and pores (by translocators) to a functional injectisome that can invade a host cell and inject effectors (Figure 1A). Recruitment of either translocator- or effector–chaperone complexes to the export apparatus depends on SctW to SctV interaction (Portaliou et al., 2017; Yuan et al., 2021). The gatekeeper complex, SctW-SepD-CesL in EPEC (Deng et al., 2005; Wang et al., 2008), and environmental signals, such as calcium (Ca) or pH changes (Matthews-Palmer et al., 2021; Gaytan et al., 2018; Pienkoss et al., 2021; Deng et al., 2005), have been associated with secretion switching from translocators to effectors at the end of the injectisome assembly. Nevertheless, the underlying molecular mechanism and roles of Ca, gatekeeper, and SctV in the course of this process are poorly understood.

In this study, we demonstrate that both the gatekeeper and the major export apparatus component SctV bind calcium in solution with high affinity (Figures 2A, 3C), at non-canonical binding site(s) that remain elusive and could be multiple (Supplementary Figures S1B, S2A). A Ca-binding site on the SctW D3 domain seems likely since mutations in the region abolish Ca-binding (Figure 2A). The SctV Ca-binding site lies within its C-domain, presumably on an interface formed by adjacent protomers, as suggested by a single-point mutation that concomitantly abolished oligomerization (Yuan et al., 2021) and Ca binding (Figure 3C).

Ca binding is not needed for anchoring the gatekeeper to the export apparatus but determines the affinity of this association (Figure 2B). We have demonstrated that SctV is the membrane receptor of the gatekeeper and have dissected the requirements for two distinct binding events of reduced and increased affinity, establishing an interplay between Ca binding, gatekeeper, and SctV. Low Ca or inability of the gatekeeper to bind Ca dictates relatively low-binding affinity on SctV, for which the SctV N-domain suffices (Figures 2B, 3A). Ca binding on the gatekeeper transforms its association with SctV to a higher affinity one (Figure 2B). The requirements for this binding differ from those needed for the lower affinity one. The SctV N-domain does not suffice anymore, nor does SctV alone; the presence of both the SctV C-domain and the injectisome is now required (Figure 3A). Combining our results with previously identified SctW-binding sites on the SctV C-domain (Portaliou et al., 2017), we conclude that, under high Ca concentration, the gatekeeper binds additionally on the SctV C-domain. In agreement, a single point mutation within the SctV C-domain freezes the gatekeeper-SctV association to a relatively high affinity one and disrupts the crucial cross-talk between Ca, gatekeeper, and SctV (Figure 4D).

Conformational changes in the SctW D3-domain, previously identified as a translocator receptor and essential for secretion switch (Portaliou et al., 2017; Archuleta and Spiller, 2014), might promote binding on the SctV C-domain in high Ca concentration. As changes in the dynamics of SctW were not detected (Supplementary Table S1), we assume that such changes involve domain motions/relative orientation. In support of our hypothesis, mutations/small deletions on the D3-domain freeze the gatekeeper–SctV association to one conformation of relatively high or low affinity (Figure 2B). Slight changes in the dynamics of the SctV C-domain upon Ca binding have been recorded (Figure 4A). These changes, supported by interactions with the SctRSTU or/and inner membrane ring components like SctD (Majewski et al., 2020; Brianceau et al., 2025; Marcos-Vilchis et al., 2025; Miletic et al., 2021), may translate through long range effects in larger changes, facilitating conformational changes on the SctV ring that enable relatively high affinity binding of the SctW D3-domain on the SctV C-domain.

SctV, and more specifically its C-domain, is the single effector receptor (Figure 3B; Yuan et al., 2021). Effectors retain an unwavering, relatively high affinity for their receptor, irrespective of injectisome components or Ca conditions, as long as the gatekeeper is not present (Figures 1D, 3B). On the other hand, translocators can bind with relatively low affinity to the SctV-N part; neither the gatekeeper nor other injectisome components nor Ca are needed for this binding (Figures 1D, 3B). However, for higher-affinity translocator binding, a bipartite Ca-gatekeeper-SctV receptor is needed. The fact that effectors can approach their relatively high-affinity SctV C-domain binding site when the gatekeeper is absent or not bound with Ca suggests that a gatekeeper that binds with increased affinity may sterically hinder the effector site, thereby favoring translocator targeting and secretion. Lack of Ca binding releases the gatekeeper from its previous relatively high-affinity binding site, without necessarily disengaging it from its relatively low binding site on the SctV N-part as well. This immediately permits increased affinity effector targeting on the SctV C-domain and secretion (Figures 1C,D, 3A). By determining the affinity of the gatekeeper for SctV, Ca commands the affinity of translocators/effectors for the channel and becomes a ruling factor for substrate targeting, thereby secretion, acting as a secretion switch (Figure 1C).

**Figure 5:**
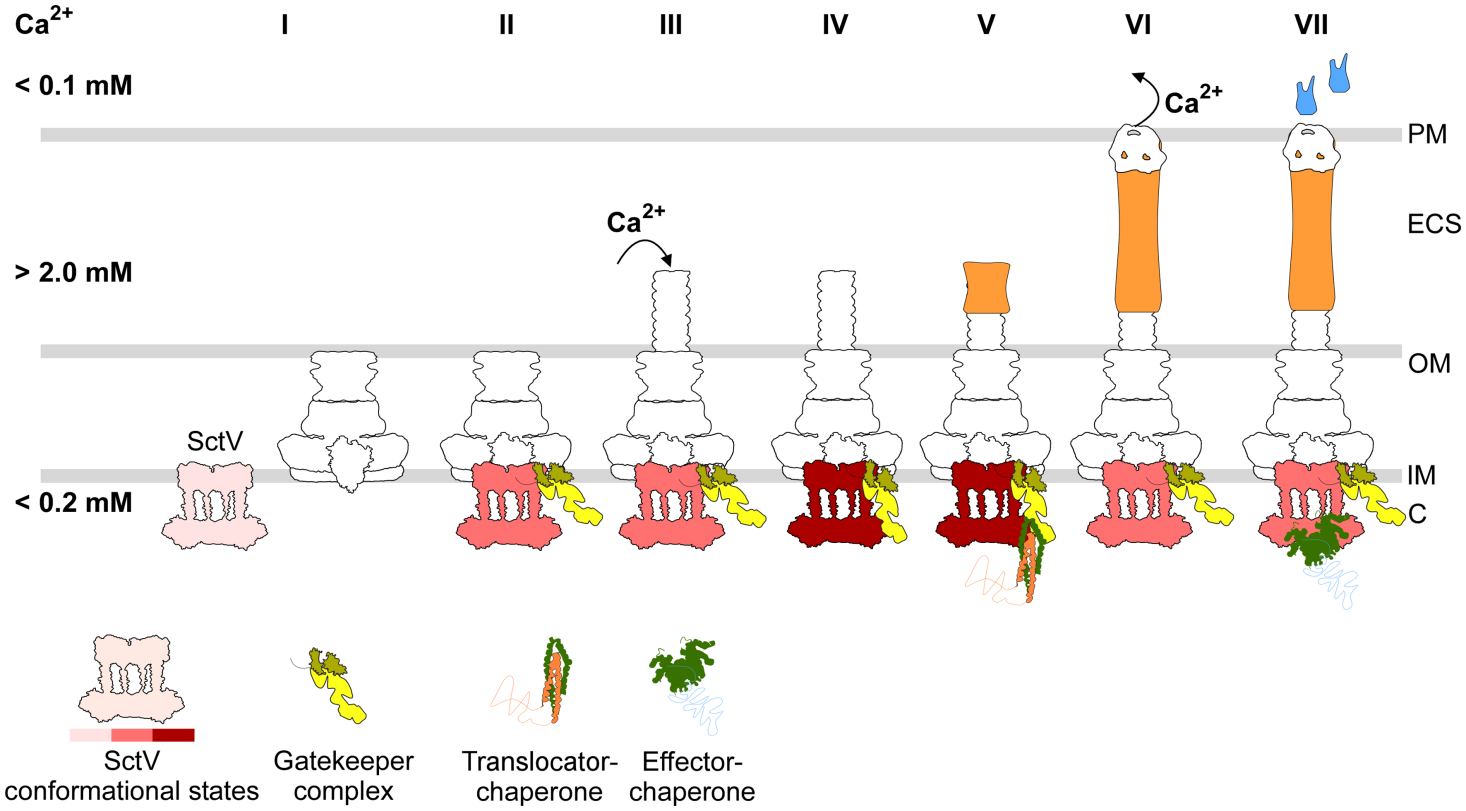
Cartoon illustration of the calcium-mediated T3S switch model in EPEC (see text for details). Pink-red: Different shades indicate different conformational states of the SctV; yellow: the gatekeeper complex (SctW-SepD-CesL); green: substrate chaperone; orange: translocator; blue: effector. C: Bacterial cytoplasm; IM: inner membrane; OM: outer membrane; ECS: extracellular space; PM: host plasma membrane.

Based on the study of others and our observations, the role of Ca, SctW, and SctV in T3SS can be combined into one unifying scheme (Figure 5): (i) Following secretion by the Sec system (Tsirigotaki et al., 2017), SctV self-assembles into a nonameric ring structure (Yuan et al., 2021; Kuhlen et al., 2021; Majewski et al., 2020) that retains a Ca-agnostic conformation (indicated by pink) and presumably prevents substrate leakage at the periplasm. The SctV_9_ slides in the IM plane toward the rest of the basal body/export apparatus that assemble independently (Brianceau et al., 2025; Deng et al., 2017). (ii) Once incorporated, the SctV conformation changes (darker pink) to a Ca-responsive one. Nevertheless, as Ca remains of <0.2 mM, the gatekeeper can only bind at the relatively low-affinity SctV-N-site, and early substrates are secreted. (iii) The needle formation likely establishes a Ca- influx from the gut epithelium/extracellular space (>2 mM) to the bacterial cytoplasm (<0.2 mM) that increases the local Ca concentration on the cytoplasmic side of the channel (Jairaman and Prakriya, 2024; Lee and Auersperg, 1980). (iv) High Ca secures formation of a bipartite Ca–SctW–SctV receptor, occupying the SctV–C-domain. (v) As a result, translocators are targeted with increased affinity and become secreted. (vi) Embedment of the translocator pore into the host cell membrane reverses the Ca flux (Lee and Auersperg, 1980), thereby releasing the gatekeeper from its relatively high affinity SctV site. (vii) As a result, the SctV C-domain becomes available for effectors that bind with increased affinity and secrete, infecting the host cell.

## Data Availability

The original contributions presented in the study are included in the article/Supplementary material, further inquiries can be directed to the corresponding author.

## Footnotes

1
https://web.expasy.org/protparam/

2
https://www.bioinformatics.org/calpred/

## References

[ref1] Archuleta T. L. Spiller B. W. (2014). A gatekeeper chaperone complex directs translocator secretion during type three secretion. PLoS Pathog. 10:e1004498. doi: 10.1371/journal.ppat.1004498, 25375170 PMC4222845

[ref2] Barkalita L. Portaliou A. G. Loos M. S. Yuan B. Karamanou S. Economou A. (2020). A reporter system for fast quantitative monitoring of type 3 protein secretion in enteropathogenic *E. coli*. Microorganisms 8:1786. doi: 10.3390/microorganisms8111786, 33202599 PMC7696366

[ref3] Berger C. Ravelli R. B. G. López-Iglesias C. Kudryashev M. Diepold A. Peters P. J. (2021). Structure of the *Yersinia* injectisome in intracellular host cell phagosomes revealed by cryo FIB electron tomography. J. Struct. Biol. 213:107701. doi: 10.1016/j.jsb.2021.107701, 33549695

[ref4] Botteaux A. Sory M. P. Biskri L. Parsot C. Allaoui A. (2009). MxiC is secreted by and controls the substrate specificity of the *Shigella flexneri* type III secretion apparatus. Mol. Microbiol. 71, 449–460. doi: 10.1111/j.1365-2958.2008.06537.x, 19017268

[ref5] Brianceau C. Wimmi S. Kronenberger T. Diepold A. (2025). Continuous exchange of an inner-membrane ring component is required for assembly and function of the type III secretion system. Nat. Commun. 16:9889. doi: 10.1038/s41467-025-65973-9, 41213942 PMC12603124

[ref6] Burkinshaw B. J. Souza S. A. Strynadka N. C. (2015). Structural analysis of SepL, an enteropathogenic *Escherichia coli* type III secretion-system gatekeeper protein. Acta Crystallogr. F Struct. Biol. Commun. 71, 1300–1308. doi: 10.1107/S2053230X15016064, 26457522 PMC4601595

[ref7] Cunningham K. Lill R. Crooke E. Rice M. Moore K. Wickner W. . (1989). SecA protein, a peripheral protein of the *Escherichia coli* plasma membrane, is essential for the functional binding and translocation of proOmpA. EMBO J. 8, 955–959. doi: 10.1002/j.1460-2075.1989.tb03457.x, 2542028 PMC400896

[ref8] Dasgupta N. Lykken G. L. Wolfgang M. C. Yahr T. L. (2004). A novel anti-anti-activator mechanism regulates expression of the *Pseudomonas aeruginosa* type III secretion system. Mol. Microbiol. 53, 297–308. doi: 10.1111/j.1365-2958.2004.04128.x, 15225323

[ref9] Deane J. E. Roversi P. King C. Johnson S. Lea S. M. (2008). Structures of the *Shigella flexneri* type 3 secretion system protein MxiC reveal conformational variability amongst homologues. J. Mol. Biol. 377, 985–992. doi: 10.1016/j.jmb.2008.01.072, 18304577 PMC2724173

[ref10] Deng W. Li Y. Hardwidge P. R. Frey E. A. Pfuetzner R. A. Lee S. . (2005). Regulation of type III secretion hierarchy of translocators and effectors in attaching and effacing bacterial pathogens. Infect. Immun. 73, 2135–2146. doi: 10.1128/IAI.73.4.2135-2146.2005, 15784556 PMC1087438

[ref11] Deng W. Marshall N. C. Rowland J. L. McCoy J. M. Worrall L. J. Santos A. S. . (2017). Assembly, structure, function and regulation of type III secretion systems. Nat. Rev. Microbiol. 15, 323–337. doi: 10.1038/nrmicro.2017.20, 28392566

[ref12] Dickenson N. E. Zhang L. Epler C. R. Adam P. R. Picking W. L. Picking W. D. (2010). Conformational changes in IpaD from *Shigella flexneri* upon binding bile salts provide insight into the second step of type III secretion. Biochemistry 50, 172–180. doi: 10.1021/bi101365f, 21126091 PMC3130115

[ref13] Dominguez D. C. Guragain M. Patrauchan M. (2015). Calcium binding proteins and calcium signaling in prokaryotes. Cell Calcium 57, 151–165. doi: 10.1016/j.ceca.2014.12.006, 25555683

[ref14] Gaytan M. O. Monjarás Feria J. Soto E. Espinosa N. Benítez J. M. Georgellis D. . (2018). Novel insights into the mechanism of SepL-mediated control of effector secretion in enteropathogenic *Escherichia coli*. Microbiology 7:e00571. doi: 10.1002/mbo3.571, 29277965 PMC6011996

[ref15] Gouridis G. Karamanou S. Gelis I. Kalodimos C. G. Economou A. (2009). Signal peptides are allosteric activators of the protein translocase. Nature 462, 363–367. doi: 10.1038/nature08559, 19924216 PMC2823582

[ref16] Hu B. Lara-Tejero M. Kong Q. Galán J. E. Liu J. (2017). In situ molecular architecture of the *Salmonella* type III secretion machine. Cell 168, 1065–1074 e10. doi: 10.1016/j.cell.2017.02.022, 28283062 PMC5393631

[ref17] Hu J. Worrall L. J. Hong C. Vuckovic M. Atkinson C. E. Caveney N. . (2018). Cryo-EM analysis of the T3S injectisome reveals the structure of the needle and open secretin. Nat. Commun. 9:3840. doi: 10.1038/s41467-018-06298-8, 30242280 PMC6155069

[ref18] Hu J. Worrall L. J. Vuckovic M. Hong C. Deng W. Atkinson C. E. . (2019). T3S injectisome needle complex structures in four distinct states reveal the basis of membrane coupling and assembly. Nat. Microbiol. 4, 2010–2019. doi: 10.1038/s41564-019-0545-z, 31427728

[ref19] Jairaman A. Prakriya M. (2024). Calcium signaling in airway epithelial cells: current understanding and implications for inflammatory airway disease. Arterioscler. Thromb. Vasc. Biol. 44, 772–783. doi: 10.1161/ATVBAHA.123.318339, 38385293 PMC11090472

[ref20] Jensen J. L. Yamini S. Rietsch A. Spiller B. W. (2020). The structure of the type III secretion system export gate with CdsO, an ATPase lever arm. PLoS Pathog. 16:e1008923. doi: 10.1371/journal.ppat.1008923, 33048983 PMC7584215

[ref21] Johnson S. Kuhlen L. Deme J. C. Abrusci P. Lea S. M. (2019). The structure of an injectisome export gate demonstrates conservation of architecture in the core export gate between flagellar and virulence type III secretion systems. MBio 10, e00818–e00819. doi: 10.1128/mbio.00818-19 31239376 PMC6593402

[ref22] Kuhlen L. Abrusci P. Johnson S. Gault J. Deme J. Caesar J. . (2018). Structure of the core of the type III secretion system export apparatus. Nat. Struct. Mol. Biol. 25, 583–590. doi: 10.1038/s41594-018-0086-9, 29967543 PMC6233869

[ref23] Kuhlen L. Johnson S. Cao J. Deme J. C. Lea S. M. (2021). Nonameric structures of the cytoplasmic domain of FlhA and SctV in the context of the full-length protein. PLoS One 16:e0252800. doi: 10.1371/journal.pone.0252800, 34143799 PMC8213127

[ref24] Kuhlen L. Johnson S. Zeitler A. Bäurle S. Deme J. C. Caesar J. J. E. . (2020). The substrate specificity switch FlhB assembles onto the export gate to regulate type three secretion. Nat. Commun. 11:1296. doi: 10.1038/s41467-020-15071-9, 32157081 PMC7064499

[ref25] Lara-Tejero M. Kato J. Wagner S. Liu X. Galán J. E. (2011). A sorting platform determines the order of protein secretion in bacterial type III systems. Science 331, 1188–1191. doi: 10.1126/science.1201476, 21292939 PMC3859126

[ref26] Lee H. C. Auersperg N. (1980). Calcium in epithelial cell contraction. J. Cell Biol. 85, 325–336. doi: 10.1083/jcb.85.2.325, 6768754 PMC2110622

[ref27] Lill R. Cunningham K. Brundage L. A. Ito K. Oliver D. Wickner W. (1989). SecA protein hydrolyzes ATP and is an essential component of the protein translocation ATPase of *Escherichia coli*. EMBO J. 8, 961–966. doi: 10.1002/j.1460-2075.1989.tb03458.x, 2542029 PMC400897

[ref28] Majewski D. D. Lyons B. J. E. Atkinson C. E. Strynadka N. C. J. (2020). Cryo-EM analysis of the SctV cytosolic domain from the enteropathogenic *E. coli* T3SS injectisome. J. Struct. Biol. 212:107660. doi: 10.1016/j.jsb.2020.107660, 33129970

[ref29] Manisha Y. Srinivasan M. Jobichen C. Rosenshine I. Sivaraman J. (2024). Sensing for survival: specialised regulatory mechanisms of type III secretion systems in gram-negative pathogens. Biol. Rev. Camb. Philos. Soc. 99, 837–863. doi: 10.1111/brv.13047, 38217090

[ref30] Marcos-Vilchis A. Espinosa N. Alvarez A. F. Puente J. L. Soto J. E. González-Pedrajo B. (2025). On the role of the sorting platform in hierarchical type III secretion regulation in enteropathogenic *Escherichia coli*. J. Bacteriol. 207:e0044624. doi: 10.1128/jb.00446-24, 40029102 PMC11925242

[ref31] Matthews-Palmer T. R. S. Gonzalez-Rodriguez N. Calcraft T. Lagercrantz S. Zachs T. Yu X. J. . (2021). Structure of the cytoplasmic domain of SctV (SsaV) from the Salmonella SPI-2 injectisome and implications for a pH sensing mechanism. J. Struct. Biol. 213:107729. doi: 10.1016/j.jsb.2021.107729, 33774138 PMC8223533

[ref32] Miletic S. Fahrenkamp D. Goessweiner-Mohr N. Wald J. Pantel M. Vesper O. . (2021). Substrate-engaged type III secretion system structures reveal gating mechanism for unfolded protein translocation. Nat. Commun. 12:1546. doi: 10.1038/s41467-021-21143-1, 33750771 PMC7943601

[ref33] Miletic S. Goessweiner-Mohr N. Marlovits T. C. (2020). The structure of the type III secretion system needle complex. Curr. Top. Microbiol. Immunol. 427, 67–90. doi: 10.1007/82_2019_178, 31667599

[ref34] Monjaras Feria J. V. Lefebre M. D. Stierhof Y.-D. Galán J. E. Wagner S. (2015). Role of autocleavage in the function of a type III secretion specificity switch protein in *Salmonella enterica* serovar typhimurium. mBio 6, e01459–e01415. doi: 10.1128/mBio.01459-15, 26463164 PMC4620466

[ref35] Nawrotek A. Guimarães B. G. Velours C. Subtil A. Knossow M. Gigant B. (2014). Biochemical and structural insights into microtubule perturbation by CopN from *Chlamydia pneumoniae*. J. Biol. Chem. 289, 25199–25210. doi: 10.1074/jbc.M114.568436, 25056950 PMC4155683

[ref36] O'Connell C. B. Creasey E. A. Knutton S. Elliott S. Crowther L. J. Luo W. . (2004). SepL, a protein required for enteropathogenic *Escherichia coli* type III translocation, interacts with secretion component SepD. Mol. Microbiol. 52, 1613–1625. doi: 10.1111/j.1365-2958.2004.04101.x, 15186412

[ref37] Pienkoss S. Javadi S. Chaoprasid P. Nolte T. Twittenhoff C. Dersch P. . (2021). The gatekeeper of *Yersinia* type III secretion is under RNA thermometer control. PLoS Pathog. 17:e1009650. doi: 10.1371/journal.ppat.1009650, 34767606 PMC8612567

[ref38] Portaliou A. G. Tsolis K. C. Loos M. S. Balabanidou V. Rayo J. Tsirigotaki A. . (2017). Hierarchical protein targeting and secretion is controlled by an affinity switch in the type III secretion system of enteropathogenic *Escherichia coli*. EMBO J. 36, 3517–3531. doi: 10.15252/embj.201797515, 29109154 PMC5709732

[ref39] Portaliou A. G. Tsolis K. C. Loos M. S. Zorzini V. Economou A. (2016). Type III secretion: building and operating a remarkable nanomachine. Trends Biochem. Sci. 41, 175–189. doi: 10.1016/j.tibs.2015.09.005, 26520801

[ref40] Romo-Castillo M. Andrade A. Espinosa N. Monjarás Feria J. Soto E. Díaz-Guerrero M. . (2014). EscO, a functional and structural analog of the flagellar FliJ protein, is a positive regulator of EscN ATPase activity of the enteropathogenic *Escherichia coli* injectisome. J. Bacteriol. 196, 2227–2241. doi: 10.1128/JB.01551-14, 24706741 PMC4054183

[ref41] Rossi P. Xing Q. Bini E. Portaliou A. G. Clay M. C. Warren E. M. . (2023). Chaperone recycling in late-stage flagellar assembly. J. Mol. Biol. 435:167954. doi: 10.1016/j.jmb.2023.167954, 37330284 PMC10471782

[ref42] Sanchez-Garrido J. Ruano-Gallego D. Choudhary J. S. Frankel G. (2022). The type III secretion system effector network hypothesis. Trends Microbiol. 30, 524–533. doi: 10.1016/j.tim.2021.10.007, 34840074

[ref43] Schubot F. D. Jackson M. W. Penrose K. J. Cherry S. Tropea J. E. Plano G. V. . (2005). Three-dimensional structure of a macromolecular assembly that regulates type III secretion in *Yersinia pestis*. J. Mol. Biol. 346, 1147–1161. doi: 10.1016/j.jmb.2004.12.036, 15701523

[ref44] Shaulov L. Gershberg J. Deng W. Finlay B. B. Sal-Man N. (2017). The ruler protein EscP of the enteropathogenic *Escherichia coli* type III secretion system is involved in calcium sensing and secretion hierarchy regulation by interacting with the gatekeeper protein SepL. MBio 8, e01733–e01716. doi: 10.1128/mbio.01733-16, 28049143 PMC5210495

[ref45] Shen D. K. Blocker A. J. (2016). MxiA, MxiC and IpaD regulate substrate selection and secretion mode in the T3SS of *Shigella flexneri*. PLoS One 11:e0155141. doi: 10.1371/journal.pone.0155141, 27171191 PMC4865121

[ref46] Straley S. C. Plano G. V. Skrzypek E. Haddix P. L. Fields K. A. (1993). Regulation by Ca2+ in the *Yersinia* low-Ca2+ response. Mol. Microbiol. 8, 1005–1010. doi: 10.1111/j.1365-2958.1993.tb01644.x, 8361348

[ref47] Tsirigotaki A. . (2017). Protein export through the bacterial sec pathway. Nat. Rev. Micro. 15, 21–36. 10.1038/nrmicro.2016.161 27890920

[ref48] Wagner S. Grin I. Malmsheimer S. Singh N. Torres-Vargas C. E. Westerhausen S. (2018). Bacterial type III secretion systems: a complex device for the delivery of bacterial effector proteins into eukaryotic host cells. FEMS Microbiol. Lett. 365:fny201. doi: 10.1093/femsle/fny201, 30107569 PMC6140923

[ref49] Wales T. E. Engen J. R. (2006). Hydrogen exchange mass spectrometry for the analysis of protein dynamics. Mass Spectrom. Rev. 25, 158–170. doi: 10.1002/mas.20064, 16208684

[ref50] Wang D. Roe A. J. McAteer S. Shipston M. J. Gally D. L. (2008). Hierarchal type III secretion of translocators and effectors from *Escherichia coli* O157:H7 requires the carboxy terminus of SepL that binds to Tir. Mol. Microbiol. 69, 1499–1512. doi: 10.1111/j.1365-2958.2008.06377.x, 18673458

[ref51] Wimmi S. Balinovic A. Brianceau C. Pintor K. Vielhauer J. Turkowyd B. . (2024). Cytosolic sorting platform complexes shuttle type III secretion system effectors to the injectisome in *Yersinia enterocolitica*. Nat. Microbiol. 9, 185–199. doi: 10.1038/s41564-023-01545-1, 38172622 PMC10769875

[ref52] Younis R. Bingle L. E. H. Rollauer S. Munera D. Busby S. J. Johnson S. . (2010). SepL resembles an aberrant effector in binding to a class 1 type III secretion chaperone and carrying an N-terminal secretion signal. J. Bacteriol. 192, 6093–6098. doi: 10.1128/JB.00760-10, 20833800 PMC2976445

[ref53] Yu X.-J. McGourty K. Liu M. Unsworth K. E. Holden D. W. (2010). pH sensing by intracellular *Salmonella* induces effector translocation. Science 328, 1040–1043. doi: 10.1126/science.1189000, 20395475 PMC6485629

[ref54] Yu X. J. . (2018). SsaV interacts with SsaL to control the translocon-to-effector switch in the *Salmonella* SPI-2 type three secretion system. MBio 9. 10.1128/mBio.01149-18 PMC6168863 30279280

[ref55] Yuan B. Economou A. Karamanou S. (2018). Optimization of type 3 protein secretion in enteropathogenic *Escherichia coli*. FEMS Microbiol. Lett. 365. 10.1093/femsle/fny122 29800479

[ref56] Yuan B. Portaliou A. G. Parakra R. Smit J. H. Wald J. Li Y. . (2021). Structural dynamics of the functional nonameric type III translocase export gate. J. Mol. Biol. 433:167188. doi: 10.1016/j.jmb.2021.167188, 34454944

