# Supplementary material for: Calcium controls type III secretion switch through an SctV-SctW interplay
Source: Front Microbiol. 2026 May 19;17:1800077. doi: 10.3389/fmicb.2026.1800077 (PMC13231503; doi:10.3389/fmicb.2026.1800077)
Supplement: Supplementary file 1 [file Data_Sheet_1.pdf]

Supplementary Information

Calcium controls type III secretion switch through an SctV-SctW interplay

Athina G Portaliou[1], Pritam Roy[1], Rinky Parakra[1], Ana-Nicoleta Bondar[2,3], Spyridoula Karamanou[1]* and Anastassios Economou[1, †]

[1]KU Leuven, Department of Microbiology, Immunology and Transplantation, Rega Institute for Medical Research, Laboratory of Molecular Bacteriology, B-3000 Leuven, Belgium
[2]University of Bucharest, Faculty for Interdisciplinary Sciences, Str. Transilvaniei 6, Bucharest, Romania
[3]Institute for Computational Biomedicine (INM-9), Forschungszentrum Jülich, Jülich, Germany

† deceased

*For correspondence: lily.karamanou@kuleuven.be

Abbreviations

AHT:	Anhydrotetracycline
EPEC:	Enteropathogenic *E. coli*
SEC-MALS:	Size exclusion chromatography coupled to multi-angle light scattering
IMVs:	Inverted Membrane Vesicles
IPTG:	Isopropyl β-D-1-thiogalactopyranoside
T3S:	Type 3 Secretion
T3SS:	Type 3 Secretion System
MD:	Molecular Dynamics
EGTA:	Ethylene Glycol Tetra-acetic Acid
UPLC:	Ultra Performance Liquid Chromatography

Table of contents

Supplementary Figures:
Figure S1: SctW Ca- defective mutants fail to restore secretion (related to Figs 1-2)
Figure S2: SctV-E489D can bind Ca but cannot restore secretion (related to Figs 3-4)

Supplementary materials and methods:
Molecular Dynamics simulations

Supplementary Tables:
Table S1: Summary of HDX-MS experimental conditions and data (*uploaded as an Excel file*; related to Fig. 4)
Table S2: Buffers
Table S3: Antisera
Table S4: Bacterial strains
Table S5: Vectors and genetic constructs
Table S6: List of primers used for gene cloning

References

Supplementary Figures

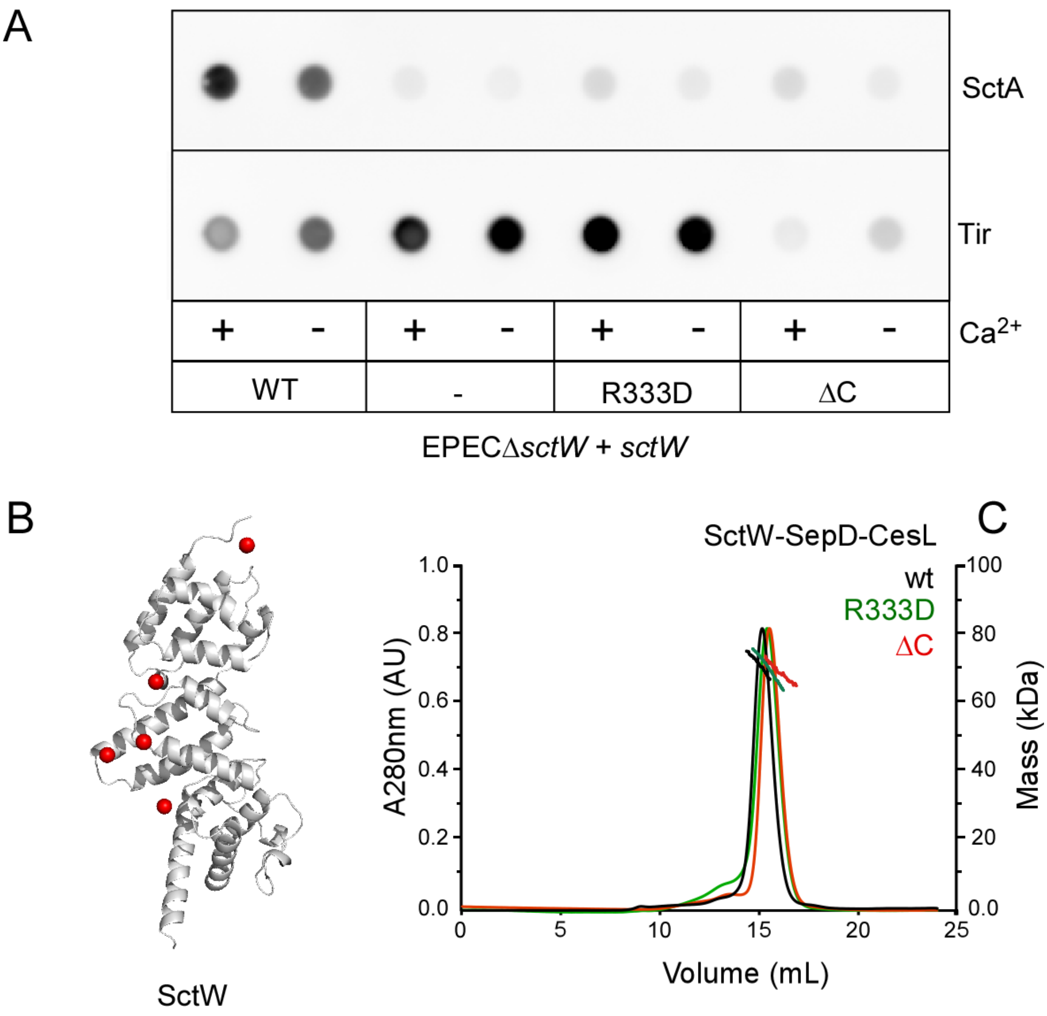

Figure S1: SctW Ca- defective mutants fail to restore secretion (related to Figs 1-2).

A. *in vivo* secretion of SctA and Tir (as indicated) by EPECΔ*sctW* cells complemented *in trans* with *sctW* or the indicated derivatives, under - / + Ca conditions. A representative experiment is shown. *n*= 3 independent repeats.
B. Calcium molecules (red) can bind on multiple SctW sites, according to Molecular Dynamics simulation analysis.
C. SEC-MALS analysis of purified His-SctW-CesL-SepD complex (or derivatives; as indicated). UV traces (left axis) and MW measurements (right axis) from representative experiments are shown. *n* = 3 independent repeats.

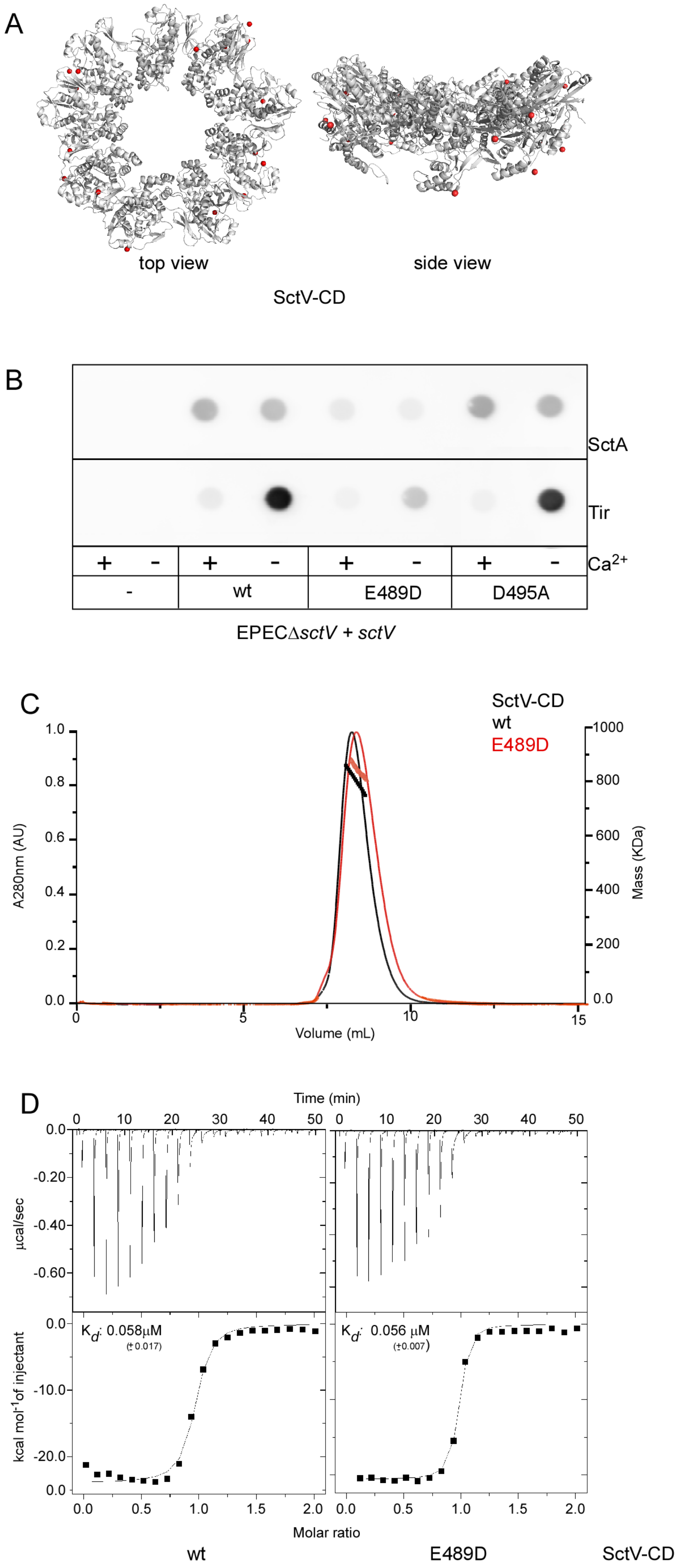
A
top view
side view
SctV-CD
B
SctA
Tir
Ca2+
+ - + - + - + -
- wt E489D D495A
EPECΔsctV + sctV
C
SctV-CD
wt
E489D
A280nm (AU)
Mass (KDa)
Volume (mL)
D
Time (min)
µcal/sec
kcal mol-1of injectant
Kd: 0.058µM (±0.017)
Kd: 0.056 µM (±0.007)
Molar ratio
wt
E489D
SctV-CD

Figure S2: SctV-E489D can bind Ca but cannot restore secretion (related to Figs 3-4).

A. Calcium molecules (red) can bind on multiple SctV C-domain sites, according to Molecular Dynamics simulation analysis.
B. *in vivo* secretion of SctA and Tir (as indicated) by EPECΔ*sctV* cells complemented *in trans* with *sctV* or the indicated derivatives, under - / + Ca conditions. A representative experiment is shown. *n*= 3 independent repeats.
C. SEC-MALS analysis of purified His-SctV-CD (wt or E489D; as indicated). UV traces (left axis) and MW measurements (right axis) from representative experiments are shown. *n* = 3 independent repeats.
D. Apparent dissociation constants (K_d) of the SctV-CD (wt or E489D; as indicated) for calcium in solution, by ITC. *n* = 3 independent repeats.

Supplementary materials and methods:

Molecular Dynamics simulations (MD):

MD simulations were performed to investigate the interaction between calcium ions and SctW or SctV-CD. SctW structure (PDB: 5C9E) and SctV-CD structure (PDB: 7OSL) were used as starting coordinates and protein side chains were considered in standard protonation states: aspartic and glutamic residues were negatively charged, arginine and lysine residues were positively charged, and histidine residues are represented as neutral-Nδ1 tautomers. Using CHARMM-GUI (Jo et al., 2008; Lee et al., 2016), proteins were solvated with TIP3P water molecules in a cubic water box with a side of 220 Å (~25 Å protein-edge distance) and neutralizing concentrations of Ca2+ and Cl2- ions, for a total of 1.016.351 atoms. Simulations were run using NAMD (Kalé, 1999; Phillips et al., 2005; Phillips et al., 2020) and CHARMM 36m force-field for protein and ions (Brooks et al., 2009; Brooks et al., 1983; MacKerell et al., 1998) and TIP3P water (Brooks et al., 1983), length of covalent bonds to H atoms were fixed using a switch function between 10-12 Å for the short-range real space interactions and smooth particle mesh Ewald summation for the Coulomb interactions (Darden, 1993; Essmann et al., 1995). We used the standard CHARMM-GUI equilibration protocol with velocity rescaling followed by production run without any constraints. During equilibration and the first 1 ns of production run we used an integration step of 1 fs. For the remaining of the production run, we used a reversible multiple timestep integration algorithm (Tuckermann, 1992) with 1 fs for the bonded forces, 2 fs for short-range non-bonded, and 4 fs for long-range non-bonded interactions. To perform the simulations at constant temperature T = 303.15K and pressure P = 1bar we used a Langevin dynamics scheme and Nosé Hoover piston (Martyna et al., 1994). We saved coordinates each picosecond. All images from the MD simulations were prepared using Visual Molecular Dynamics, VMD (Humphrey et al., 1996).

Supplementary Tables:

Table S1: Summary of HDX-MS experimental conditions and data (*uploaded as an Excel file*; related to Fig. 4).

Table S2: Buffers

**Buffer A**	50 mM Tris-HCl pH 8.0; 50mM M NaCl
**Buffer B**	50 mM Tris-HCl pH 8.0; 50 mM KCl;5mM Mg_2Cl
**Buffer C**	50 mM Tris-HCl pH 8.0; 50 mM NaCl; 50% Glycerol (storage buffer)
**Buffer D**	25 mM Tris-HCl pH 8.0; 25 M NaCl
**Quench Buffer**	8M Urea; 0.1% DDM; 5mM TCEP pH 2.5
**Buffer E**	50 mM Tris-HCl pH 8.0; 1M NaCl; 5mM Imidazole; 5% Glycerol
**Buffer F**	50 mM Tris-HCl pH 8.0; 50mM NaCl; 5mM Imidazole; 5% Glycerol
**1XM9 salts**	33.7 mM Na_2HPO_4; 22 mM KH_2PO_4; 8.55 mM NaCl; 9.35 mM NH_4Cl

Table S3: Antisera

Rabbit polyclonal antibodies against the indicated purified proteins or protein domains were raised by Davids Biotechnologie, Germany. Antibodies against T3SS-related proteins were further purified by 9 cycles of negative immuno-absorption, using membranes isolated from EPEC strains that lacked the gene of interest, i.e. for α-SctJ, membranes isolated from EPECΔ*sctJ* cells were used.

Antibody	Animal Source	Reference or commercial source

α-SctA	Rabbit	(Portaliou et al., 2017)
α-Tir	Rabbit	(Portaliou et al., 2017)
α-Rabbit IgG	Goat	Jackson ImmunoResearch Europe Ltd

Table S4: Bacterial strains

**Bacterial strain *E. coli***	**Description (gene deleted)**	**Reference/source**
DH5a	F^- *endA1 glnV44 thi-1 recA1 relA1 gyrA96 deoR nupG purB20* φ80d*lacZ*ΔM15 Δ(*lacZYA-argF*)U169, hsdR17($r_K^- m_K^+$), λ^-	Invitrogen
BL21(DE3)	*E. coli* str. B F^- *ompT gal dcm lon hsdS*$_B$($r_B^- m_B^-$) λ(DE3 [*lacI lacUV5-T7p07 ind1 sam7 nin5*]) [*malB*$^+$]$_{K\text{-}12}$(λ^S)	(Studier et al., 1990)
C41(DE3)	F^- *ompT gal dcm hsdS*$_B$($r_B^- m_B^-$)(DE3)	Lucigen (Miroux and Walker, 1996)
EPEC E2348/69	*E. coli* O127:H6 (strain E2348/69)	(Levine et al., 1978)
EPEC E2348/69 Δ*sctV*	Δ*sctV*:: *nptII*(Kan^R)	(Portaliou et al., 2017)
EPEC E2348/69 Δ*sctW*	Δ*sctW*:: *nptII*(Kan^R)	(Portaliou et al., 2017)
EPEC E2348/69 *sctV*ΔCD	*sctV*ΔCD:: *nptII*(Kan^R)	(Portaliou et al., 2017)

Table S5: Vectors and genetic constructs

Mutations were introduced to genes by Quick-Change PCR Mutagenesis, using mutagenic primer pairs and PFU Polymerase from Promega. All primers were constructed by Kaneka Eurogentec S.A., Liege. All PCR-generated plasmids were sequenced at Macrogen Europe, Amsterdam. Plasmids were transformed in DH5α cells and stored in 20% glycerol at −80ºC.

**Vector name**	**Antibiotic resistance**	**Promoter**	**Origin**	**Reference/source**
pASKIBA7	Amp	Tet	pBR322	IBA life sciences; (Guzman et al., 1995)
pETDuet-1	Amp	T7	pBR322	Novagen
pET16b	Amp	T7	pBR322	Novagen
**Gene**	**Uniprot KB accession**	**Plasmid name**	**Vector**	**Cloning strategy or source**
sctV	B7UMA7	pLMB0088	pASK IBA 7	(Portaliou et al., 2017)
sctV (E489D)	B7UMA7	pLMB2248	pASK IBA 7	E489D mutation was introduced in pLMB0088 using primers X2461 and X2462
sctV (D495A)	B7UMA7	pLMB2249	pASK IBA 7	D495A mutation was introduced in pLMB0088 using primers X2463 and X2464
His *sctV*CD (N334-675)	B7UMA7	pLMB1676	pET16b	(Portaliou et al., 2017)
His-*sctV*CD (N334-675; R535A)	B7UMA7	pLMB2142	pET16b	(Yuan et al., 2021)
His-*sctV*CD (N334-675; E489D	B7UMA7	pLMB2244	pET16b	E489D mutation was introduced in pIMBB1548 using primers X2461 and X2462
His *cesL/sepD/sctW*	B7UMA8, B7UMB2, B7UM95	pIMBB1548	pETDuet1	(Portaliou et al., 2017)
His *cesL/sepD/sctW* (R333D)	B7UMA8, B7UMB2, B7UM95	pLMB2184	pETDuet1	R333D mutation was introduced in pIMBB1548 using primers X1735 and X1736
His *cesL/sepD/sctW* (N 1-340; ΔC)	B7UMA8, B7UMB2, B7UM95	pLMB2216	pETDuet1	In pIMBB1548, a stop codon replaced G341 at *sctW* using primers X2444 and X2445

His *sctW*	B7UM95	pIMBB1305	pASK IBA 7	(Portaliou et al., 2017)
His *sctW* (R333D)	B7UM95	pIMBB1543	pASK IBA 7	(Portaliou et al., 2017)
His *sctW* (N1-340; ΔC)	B7UM95	pLMB2218	pASK IBA 7	In pIMBB1305, a stop codon replaced G341 at *sctW* using primers X2444 and X2445
His *cesAB/espA*	B7UMC4, B7UM94	pIMBB0648	pETDuet1	(Portaliou et al., 2017)
His *cesT/Tir*	Q47015, B7UM99	pIMBB1158	pETDuet1	(Portaliou et al., 2017)

Table S6: List of primers used for gene cloning

Primer Name	Forward/ Reverse	Restriction site	Gene	Sequence (5'-3') (restriction sites underlined/ linker italics/ mutation bold)
X1735	F		*sctW/*R333D	ATTGATAACGAGCAG**GAC**AGTAATACATTATTA
X1736	R		*sctW/*R333D	TAATAATGTATTACT**GTC**CTGCTCGTTATCAAT
X2444	F		*sctW*ΔC	ACATTATTAATGATT**TGA**AAAGTGATAGATTAT
X2445	R		*sctW*ΔC	ATAATCTATCACTTT**TCA**AATCATTAATAATGT
X2461	F		*sctW/*E489D	TTCATCGGCGTACAA**GAT**ACGCGTTATTTGATG
X2462	R		*sctW/*E489D	CATCAAATAACGCGT**ATC**TTGTACGCCGATGAA
X2463	F		*sctV/*D495A	ACGCGTTATTTGATG**GCC**ATCATGGAGAGAAAA
X2464	R		*sctV/* D495A	TTTTCTCTCCATGAT**GGC**CATCAAATAACGCGT

References

Brooks, B.R., Brooks, C.L., 3rd, Mackerell, A.D., Jr., Nilsson, L., Petrella, R.J., Roux, B., Won, Y., Archontis, G., Bartels, C., Boresch, S., *et al.* (2009). CHARMM: the biomolecular simulation program. J Comput Chem *30*, 1545-1614.

Brooks, B.R., Bruccoleri, R.E., Olafson, B.D., States, D.J., Swaminathan, S., and Karplus, M. (1983). Charmm - a Program for Macromolecular Energy, Minimization, and Dynamics Calculations. Journal of Computational Chemistry *4*, 187-217.

Darden, T., D. York, and L. Pedersen (1993). Particle mesh Ewald: an N x log(N) method for Ewald sums in large systems. J Chem Phys.

Essmann, U., Perera, L., Berkowitz, M.L., Darden, T., Lee, H., and Pedersen, L.G. (1995). A Smooth Particle Mesh Ewald Method. Journal of Chemical Physics *103*, 8577-8593.

Guzman, L.M., Belin, D., Carson, M.J., and Beckwith, J. (1995). Tight regulation, modulation, and high-level expression by vectors containing the arabinose PBAD promoter. J Bacteriol *177*, 4121-4130.

Humphrey, W., Dalke, A., and Schulten, K. (1996). VMD: visual molecular dynamics. J Mol Graph *14*, 33-38, 27-38.

Jo, S., Kim, T., Iyer, V.G., and Im, W. (2008). CHARMM-GUI: a web-based graphical user interface for CHARMM. J Comput Chem *29*, 1859-1865.

Kalé, L., R. Skeel, M. Bhandarkar, R. Brunner, A. Gursoy, N. Krawetz, J. Phillips, A. Shinozaki, K. Varadarajan, and K. Schulten (1999). NAMD2: greater scalability for parallel molecular dynamics. J Comput Phys *151:283-312*.

Lee, J., Cheng, X., Swails, J.M., Yeom, M.S., Eastman, P.K., Lemkul, J.A., Wei, S., Buckner, J., Jeong, J.C., Qi, Y., *et al.* (2016). CHARMM-GUI Input Generator for NAMD, GROMACS, AMBER, OpenMM, and CHARMM/OpenMM Simulations Using the CHARMM36 Additive Force Field. J Chem Theory Comput *12*, 405-413.

Levine, M.M., Bergquist, E.J., Nalin, D.R., Waterman, D.H., Hornick, R.B., Young, C.R., and Sotman, S. (1978). Escherichia coli strains that cause diarrhoea but do not produce heat-labile or heat-stable enterotoxins and are non-invasive. Lancet *1*, 1119-1122.

MacKerell, A.D., Bashford, D., Bellott, M., Dunbrack, R.L., Evanseck, J.D., Field, M.J., Fischer, S., Gao, J., Guo, H., Ha, S., *et al.* (1998). All-atom empirical potential for molecular modeling and dynamics studies of proteins. J Phys Chem B *102*, 3586-3616.

Martyna, G.J., Tobias, D.J., and Klein, M.L. (1994). Constant-Pressure Molecular-Dynamics Algorithms. Journal of Chemical Physics *101*, 4177-4189.
Miroux, B., and Walker, J.E.J.J.o.m.b. (1996). Over-production of proteins in Escherichia coli: mutant hosts that allow synthesis of some membrane proteins and globular proteins at high levels. *260*, 289-298.
Phillips, J.C., Braun, R., Wang, W., Gumbart, J., Tajkhorshid, E., Villa, E., Chipot, C., Skeel, R.D., Kale, L., and Schulten, K. (2005). Scalable molecular dynamics with NAMD. J Comput Chem *26*, 1781-1802.
Phillips, J.C., Hardy, D.J., Maia, J.D.C., Stone, J.E., Ribeiro, J.V., Bernardi, R.C., Buch, R., Fiorin, G., Henin, J., Jiang, W.*, et al.* (2020). Scalable molecular dynamics on CPU and GPU architectures with NAMD. J Chem Phys *153*, 044130.
Portaliou, A.G., Tsolis, K.C., Loos, M.S., Balabanidou, V., Rayo, J., Tsirigotaki, A., Crepin, V.F., Frankel, G., Kalodimos, C.G., Karamanou, S.*, et al.* (2017). Hierarchical protein targeting and secretion is controlled by an affinity switch in the type III secretion system of enteropathogenic Escherichia coli. The EMBO journal *36*, 3517-3531.
Studier, F.W., Rosenberg, A.H., Dunn, J.J., and Dubendorff, J.W. (1990). Use of T7 RNA polymerase to direct expression of cloned genes. Methods Enzymol *185*, 60-89.
Tuckermann, M., B. J. Berne, and G. J. Martyna (1992). Reversible multiple time scale molecular dynamics. J Chem Phys *97:1990-2001.*
Yuan, B., Portaliou, A.G., Parakra, R., Smit, J.H., Wald, J., Li, Y., Srinivasu, B., Loos, M.S., Dhupar, H.S., Fahrenkamp, D.*, et al.* (2021). Structural Dynamics of the Functional Nonameric Type III Translocase Export Gate. Journal of molecular biology *433*, 167188.
